# Are personalities genetically determined? Inferences from subsocial spiders

**DOI:** 10.1186/s12864-019-6172-5

**Published:** 2019-11-22

**Authors:** Jessica Purcell, Jonathan N. Pruitt

**Affiliations:** 1 Department of Entomology, University of California Riverside, 900 University Ave, Riverside, CA 92521 USA; 2 Department of Psychology, Neuroscience and Behaviour, McMaster University, 1280 Main Street West, Hamilton, ON L8S 4K1 Canada

**Keywords:** Social spiders, Boldness, Animal personality, RADseq, Genome assembly, Linkage map

## Abstract

**Background:**

Recent research has revealed that polymorphic behavioral strategies shape intra-and interspecific interactions and contribute to fitness in many animal species. A better understanding of the proximate mechanisms underlying these behavioral syndromes will enhance our grasp this phenomenon. Spiders in the genus *Anelosimus* exhibit inter-individual behavioral variation on several axes: individuals have consistent responses to stimuli (e.g. bold vs. shy individuals) and they are subsocial (exhibiting extended maternal care and sibling cooperation) across most of their range, but they sometimes form permanent social groups in northern temperate regions. Here, we seek genetic variants associated with boldness and with social structure in a socially polymorphic population of the spider *Anelosimus studiosus.* We also develop preliminary genomic resources, including a genome assembly and linkage map, that support this and future genomic research on this group.

**Results:**

Remarkably, we identify a small genomic scaffold (~ 1200 bp) that harbors seven single nucleotide polymorphisms (SNPs) associated with boldness. Moreover, heterozygotes are less common than expected based on Hardy-Weinberg equilibrium, suggesting that either assortative mating or selection against heterozygotes may be occurring in this system. We find no loci significantly associated with social organization. Our draft genome assembly allows us to localize SNPs of interest in this study and to carry out genetic comparisons with other published genomes, although it remains highly fragmented.

**Conclusions:**

By identifying a locus associated with a well-studied animal personality trait, this study opens up avenues for future research to link behavioral studies of animal personality with genotype and fitness.

## Background

Much of classical behavioral ecology has been led by optimality models, which tend to view inter-individual variation as statistical noise [17]. Nowadays, however, researchers recognize that many species harbor individual variants with highly consistent responses to different stimuli [50]. These inter-individual differences, sometimes referred to as ‘personalities’, modulate within- and between-species interactions, including competition, predator defense, and mating strategy [53]. Although repeatable personality differences have been documented in animals ranging from arthropods to mammals and are known to be heritable in some systems [19], we still know little about the specific molecular mechanisms underlying the maintenance of this variation in most systems [32]. While some behavioral traits are generated through epigenetic mechanisms, the observation that personality can be heritable suggests that genetic variation in these traits is present [19].

Recent advances in sequencing technology provide unprecedented opportunities to develop genomic resources for non-model systems and allow researchers to seek genetic associations with a broad suite of phenotypic traits, including behaviors [9]. The application of genome-wide association studies (GWAS) to animal personality traits has lagged behind other phenotypic traits, due in part to the general assumption that behaviors are either entirely phenotypically plastic at one extreme or highly polygenic at the other [51]. So far, much of our understanding of the heritability of behavioral traits comes from quantitative genetic studies of captive rodent populations (reviewed by [51]), and, in these and other systems, bold versus shy or aggressive versus docile strategies appear to have a substantial genetic component [19]. However, studies of rodents and other organisms in natural populations reveal that gene-by-environment interactions can profoundly influence behavioral phenotypes (reviewed by [34]), supporting the importance of studying behavioral traits in natural populations as well. These quantitative genetic approaches are valuable in revealing the role of genetic inheritance in shaping behavioral variation; however, we are now able to take these studies a step further and ask what genetic architecture underlies personality traits.

The spider genus *Anelosimus* harbors a broad range of social strategies, from highly “social” species that live in large groups throughout their life cycle, to “subsocial” species that exhibit extended maternal care and cooperation among subadult siblings, to solitary species that disperse early in development [3]. In recent years, this group has been the subject of much research on inter-individual behavioral variation, with studies carried out on both subsocial and highly social species (e.g. [39, 40]). *Anelosimus studiosus* is among the most broadly distributed species in the genus, with its range extending from the northeastern United States through the Andes to Patagonia [1]. Across most of its range, this species is subsocial, but some larger multifemale social groups form in northern populations [20, 26, 27]. Colony social structure and personality are independent traits, such that bold and shy individuals may live in either social or solitary colonies [38]. Pruitt and Goodnight [38] showed that the composition of spider personalities in social colonies influenced their survival and reproduction, and that the optimal composition depended upon their environment. Thus, while individual personality is not causally linked with sociality in this species, the ratio of types of individuals within colonies is a major determinant of a group’s success.

In the present study, we develop genomic resources to support the integration of research on social evolution and animal personality with genetics. We pursue four goals. We first assemble a preliminary genome (goal 1) and high-density linkage map (goal 2) for *A. studiosus.* Using double digest Restriction-site Associated DNA sequencing (ddRADseq), we then carry out a genome-wide association study to identify genomic regions associated with spider personality (goal 3) and with colony social structure (goal 4). Identifying the genetic basis of well-studied behaviors will open new avenues for research on the mechanisms underlying selection for alternative behavioral strategies in *A. studiosus* and perhaps the broader *Anelosimus* genus.

## Results

### Genome assembly

Based on our draft assembly, the *A. studiosus* genome is 2.22 Gpb with a GC content of 27.95%. The genome assembly N50 is 4622 bp; the maximum scaffold size is 78,878 bp. Using the kmercountexact.sh function in BBMap [14], we estimate that the total haploid genome size is approximately 1.58 Gbp. The difference in estimated genome size and assembled genome size could indicate that our genome assembly includes some duplicated variant regions; alternatively, the ancient genome duplication present in spiders [48] could affect the precision of kmer-based estimates of genome size.

### Linkage map assembly

**Fig. 1:**
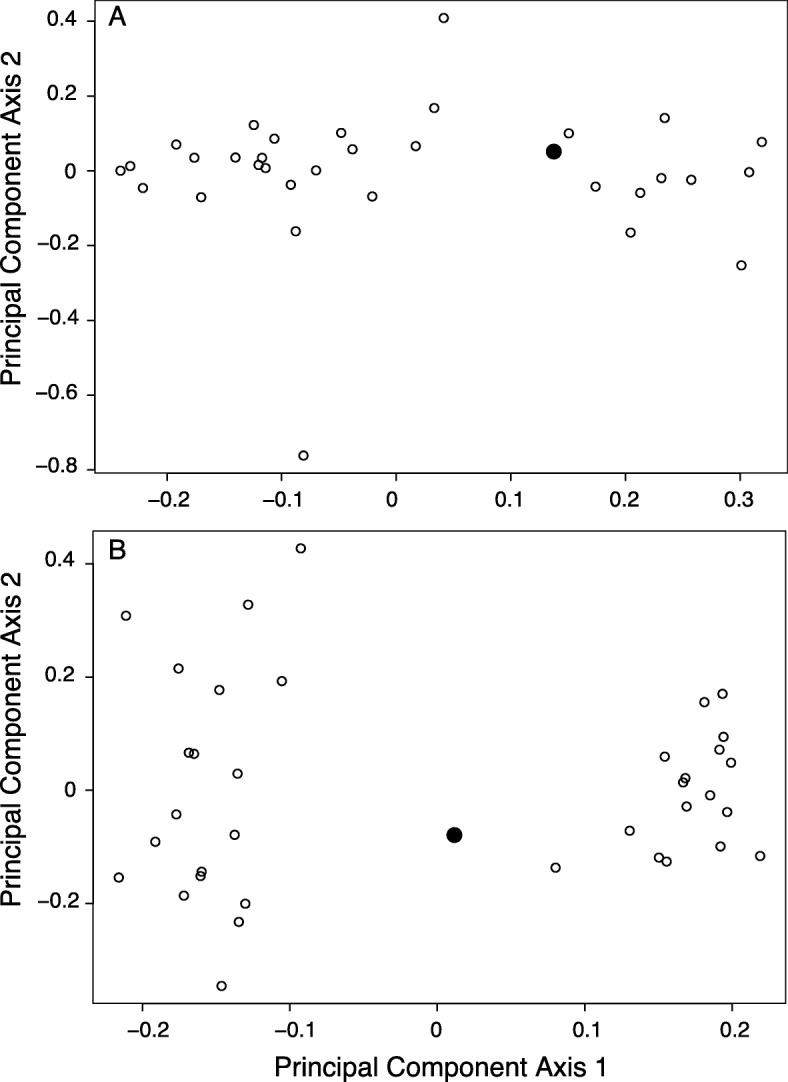
Principal component analyses of genetic structure between mother and offspring from family P1 (**a**) and family P2 (**b**) reveal a pattern consistent with multiple paternity of offspring. In both cases, the mother (enlarged black dot) is positioned between two offspring clusters (open dots) that are separated along principal component axis 1. For a follow-up assessment of relatedness (Additional file 1: Figure S2), we consider individuals with PC axis 1 values less than the mother to be putative full sib group 1, and those with values greater than the mother to be full sib group 2

Through maternal linkage maps for two families, we were able to place 284 and 541 markers in linkage groups containing five or more loci for families P1 and P2, respectively (Additional file 1: Tables S2, S3). We excluded small linkage groups (fewer than five loci) from further analyses (P1: 399 additional markers were on 301 small linkage groups, most of which contained a single locus; P2: 175 additional markers were on 135 small linkage groups). We obtained 24 and 19 linkage groups for families P1 and P2, respectively. Based on karyotypes, *Anelosimus* species have a haploid chromosome number of 12; females have 24 chromosomes (sex chromosomes are X_1_X_1_X_2_X_2_), while males have 22 chromosomes (sex chromosomes are X_1_X_2_O [5];). Thus, our linkage groups are oversplit, which is a common outcome for RADseq-based linkage maps produced with fewer than 50 offspring (Pers. Obs.). In total, 16 scaffolds harboring 28 SNPs overlapped between the two linkage maps on informative linkage groups, allowing us to determine several syntenic regions of the linkage maps (Additional file 1: Figure S1). During the linkage map assembly process, we noticed that the offspring groups appeared to have unexpected genetic structure. By carrying out principal component analyses (PCA) on offspring and the mother from both families, we were able to infer that both mothers probably mated with two different males (Fig. 1). We confirmed this by investigating relatedness patterns across a combined dataset containing spiders from our linkage mapping and association studies (Additional file 1: Figure S2). The average relatedness between individuals in putative half-sib groups was approximately half the relatedness between putative full siblings and mother/offspring pairs, which supports this inference.

### Association study

**Fig. 2:**
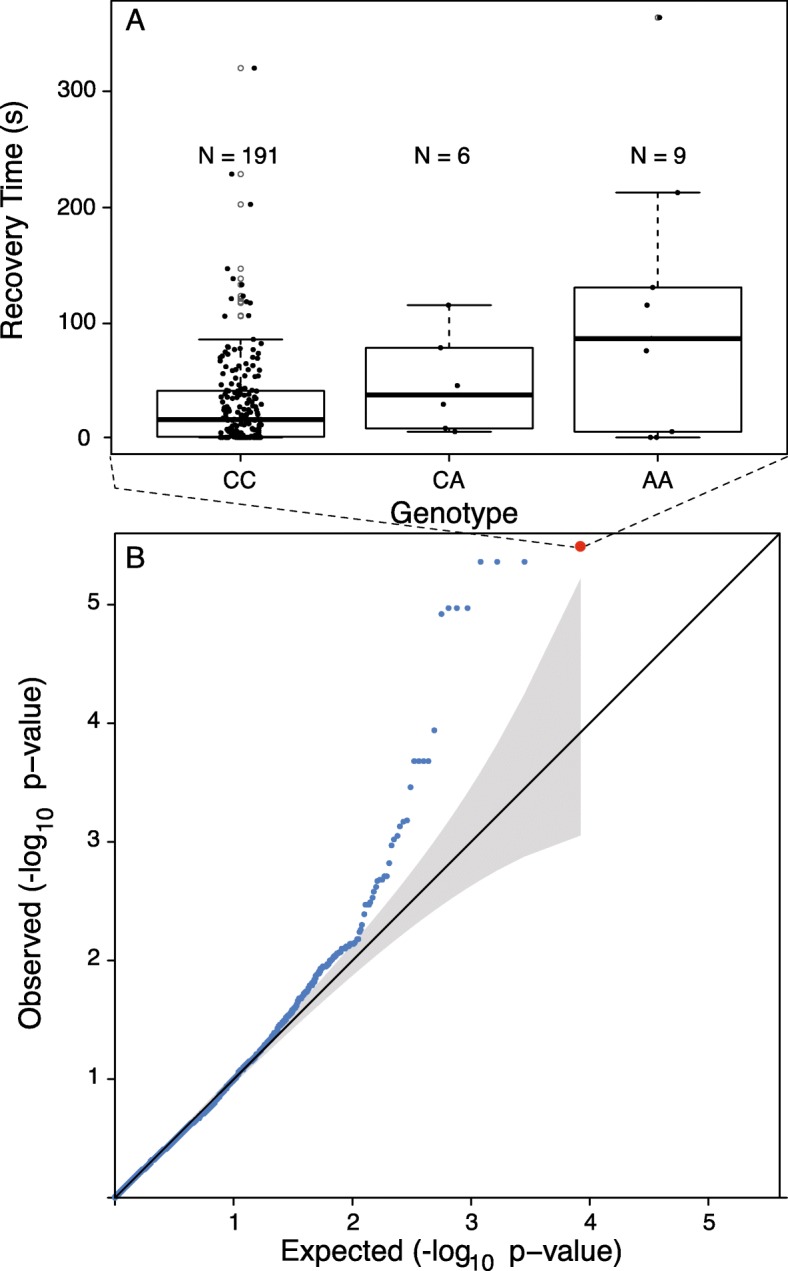
Comparison of boldness score by genotype (**a**) in individuals at the most highly associated locus of seven highly significant SNPs on scaffold 422,272 (enlarged red dot, B). The outlier loci on the boxplots are shown with light grey open circles, while all data points (including outliers) are shown as jittered, black filled circles. The QQ plot (**b**) compares the expected and observed *p*-values with the grey bar indicating 95% confidence intervals, revealing that the observed associations exhibit more significant p-values than expected by chance

**Fig. 3:**
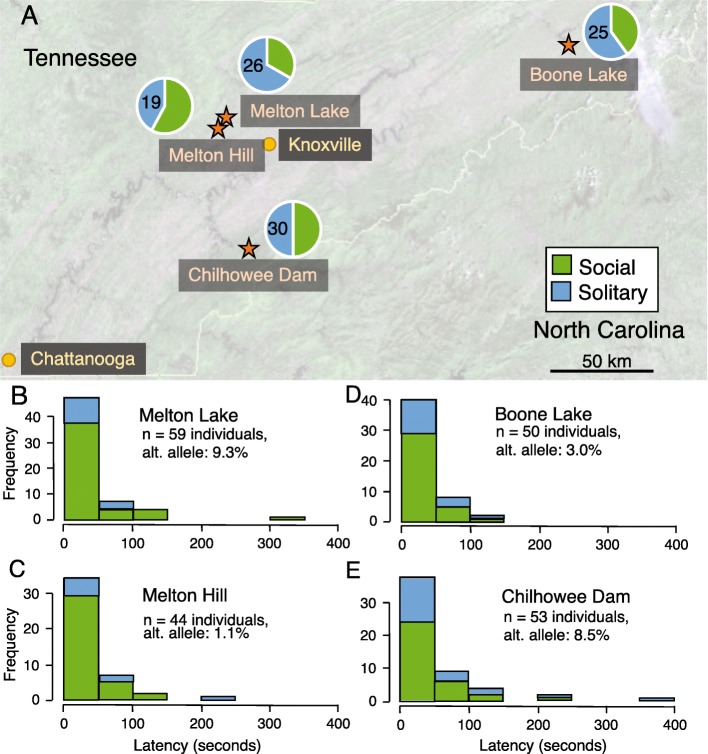
The four sampling localities in eastern Tennessee are shown with orange stars (**a**), and the total number of nests collected that were either solitary (blue) or social (green) are shown with pie charts. The distribution of latency to move after perturbation (a measure of boldness) is shown for individuals from each of the four populations (**b**: Melton Lake, **c**: Melton Hill, **d**: Boone Lake, and **e**: Chilhowee Dam). Individuals that moved quickly (low latency score) are bold, whereas individuals with a slow response (high latency score) are shy. There was no association between nest type (social or solitary) and boldness; populations differed in the frequency of the alternate allele at the locus that is most strongly associated with boldness

We identified seven tightly linked markers that are strongly associated with boldness (*p* < = 1.06 × 10^− 5^; Fig. 2). These are all found on a small scaffold (1239 bp) in the *A. studiosus* genome assembly, and explain approximately 11% of the variance in boldness. The presence of both homozygotes and of heterozygotes and confirmation that the depth of coverage did not differ systematically across each genotype suggest that these genetic variants are not the result of copy number variation in our sample. We also note that both the reference and the alternate allele for the associated SNPs were present in all four populations, despite the rarity of the alternate allele (Fig. 3). We detect a total of five distinct genotypes at the markers associated with boldness on this scaffold: the majority of individuals were homozygous for the reference allele at all seven markers, the six heterozygous individuals were heterozygous at all markers, and eight out of nine individuals homozygous for the alternative allele (Fig. 2) were consistent at all markers. We detected one individual from Melton Hill that was heterozygous at three markers, and homozygous for the reference allele for the remaining four, but we note that the depth of coverage was relatively low (less than 10x) for the heterozygous markers in this individual. We also detected one individual from Melton Lake that was homozygous for the alternative allele at one marker and homozygous for the reference allele at the remaining markers, with a depth of coverage greater than 100 for all loci in this scaffold. Taken together, these results suggest that there are two major haplotypes on this scaffold, but that recombination may occur, breaking apart the multi-SNP haplotypes occasionally.

Alignment to the house spider *Parasteatoda tepidariorum* revealed a partial hit of this scaffold to unplaced genomic scaffold with an e-value of 0.001 (Table 1). We then used blastn to align the focal *A. studiosus* scaffold to the broader NCBI database. Here, the top hit was to the *Argiope argentata* pyriform spidroin 1 gene (which encodes a silk protein). Since the sequence partially hit a coding region of a spider gene, we used blastx to examine hits to proteins (Table 1). Given the discovery that the top hit matches a transposable element in the ant *Lasius niger*, we suggest that the genomic region associated with boldness may be in a transposable element that is present in different fragments in the genomes of related spiders and other arthropods. We did not find any RADtags associated with the social structure of the colonies.

**Table 1:** NCBI blast query results based on search

Query	Top Hit(s)	Total Score	Query Cover	E value	% Identical
Blastn to *Parasteatoda tepidariorum* 2.0	Ptep_2.0 Scf5bK3_1364	50.9	11%	0.001	72.34%
Blastn to genbank	*Argiope argentata* pyriform spidroin 1 gene	52.7	22%	0.041	67.03%
Blastx to genbank	(1) Transposable element tc3 transposase (*Lasius niger*)	119	45%	10^−17^	37.50%
	(2) Hypothetical protein X975_20604 (*Stegodyphus mimosarum*)	114	36%	3 × 10^−16^	53.62%

## Discussion

Research on social spiders has given rise to broad insights about social evolution, sex ratio theory, and animal personality [2, 4, 6–8, 22–24, 39], but they have lagged behind other organisms in the development and application of genomic resources (but see [37, 49]). We identify multiple variants in a small genomic region that are strongly associated with boldness in the spider *Anelosimus studiosus* (Fig. 2)*,* and we develop a preliminary genome assembly and linkage map that will facilitate future genomic studies of this genus (Additional file 1: Figure S1, Tables S2, S3). Previous studies demonstrated that highly repeatable inter-individual behavioral variants, including boldness, were strongly heritable in *A. studiosus* [38, 41]. This study takes our understanding of this trait a step further by identifying a putative genetic basis.

We discovered that SNPs associated with boldness all occurred on a single scaffold in our dataset. This scaffold also contained four SNPs that were not associated with boldness. Using one representative SNP with the most significant association to boldness, we showed that homozygotes for the alternative allele are the most likely to have a long duration of the death feign response, indicative of a shy individual, heterozygotes are intermediate, and homozygotes for the reference allele have the lowest score (indicative of bold individuals), although there is variation in behavioral response in all categories. We note that even traits that are strongly affected by genotype may also be influenced by the environment (e.g. via gene-by-environment interactions mediated by epigenetic mechanisms) or by other loci of minor effect. The alternative allele was present in all four sampled populations. Interestingly, we observed a deviation from Hardy-Weinberg equilibrium in this marker, with fewer heterozygous individuals than expected given the allele frequency (Chi-squared = 16.58, *p* = 0.00025). This pattern could be explored by seeking fitness differences between homozygous and heterozygous individuals at this locus. We speculate that having an intermediate boldness level may be disadvantageous in this system. Alternatively, assortative mating based upon behavioral syndrome could also account for the relative scarcity of heterozygotes.

The scaffold harboring the boldness-associated loci had marginal alignments to the house spider *Parasteatoda tepidariorum* genome and to *Argiope argentata* pyriform spidroin 1 gene, which encodes a silk protein (Table 1). Given its position in an *A. argentata* coding region, we translated our DNA sequence and reran a protein search. This yielded a well-supported hit to a transposase from the ant *Lasius niger* (as well as a secondary hit to a hypothetical protein in the spider *Stegodyphus mimosarum*). Given this finding, we speculate that our scaffold may overlap with a transposable element in the *A. studiosus* genome. Transposons often take on a regulatory role in the genome [16], however, our data do not yet allow us to explore downstream effects on gene expression. We for now predict that variation at this genomic region will be associated with different gene regulatory networks.

We uncovered no genetic markers associated with colony social structure in this sample. Given our reduced representation sequencing approach, this does not rule out a genetic basis for social group formation in this species, since we sampled only a small subset of the genome.

Another outcome of this project is the discovery that *Anelosimus* genomes have challenging features, including large size and low GC content. The genome size and composition are in line with other published spider genomes [46, 48]. The repetitive nature makes genome assembly challenging, although recent innovations in sequencing and long-range genome scaffolding approaches will facilitate substantial improvements. Despite the highly fragmented nature of the genome assembly presented here, which was based entirely on short insert Illumina sequencing, being able to align RADtags to a genome for the linkage mapping and association studies improved our ability to compare maps (Additional file 1: Figure S1) and to determine where SNPs of interest were found in other species’ genomes (Table 1). In general, the outcome of population genetic analyses using reduced representation genomic sequencing methods is enhanced by alignment to genome assemblies, even low quality ones [47]. The linkage maps will, in turn, be useful in scaffolding a more contiguous genome sequence in the future. The linkage mapping project also revealed the unexpected pattern of multiple paternity in two families (Fig. 1), which contradicts a previous study in this species that detected first-male sperm precedence [28]. Their study investigated the outcome of captive matings when different males were introduced over the course of several days. Although our sample is small, we sequenced field-collected brood, and differences in the timing and outcome of mating in natural conditions may account for the conflicting results.

## Conclusion

Here, we take initial steps in developing genomic resources for a broadly useful behavioral model for the study of animal personality and social evolution. We propose several novel studies that could follow up on our preliminary results. The discovery of a genomic region that is strongly associated with boldness opens a variety of new research directions at the intersection of animal behavior and population genomics. There remains much to be discovered.

## Methods

### Genome and linkage map

In May 2014, we sampled an *A. studiosus* female subadult from a nest containing multiple females at Melton Hill Dam, Tennessee (35°89′N, 84°30′W) for whole genome sequencing. In June 2014, we sampled two families including a mother and her offspring (37 and 43 spiderlings, respectively) from solitary nests, also from Melton Hill Dam, in order to construct high-density genetic linkage maps.

### Association study

In May 2014, we sampled 226 *A. studiosus* individuals from single- and multi-female colonies at four populations in Tennessee: Chilhowee Dam, Melton Hill, Melton Lake and Boone Lake (Fig. 3). *Anelosimus studiosus* spiders generally nest in vegetation along the water’s edge, and they tend to have a patchy distribution [29, 45]. Thus, we sampled nests opportunistically by boat and/or on foot at each site. These populations harbor spiders using two alternative social strategies. Some individuals form multi-female social groups, while others exhibit the more classical subsocial strategy of dispersing and founding independent nests before maturity [45]. These spiders usually live for approximately 1 year, with mature individuals producing offspring in the late spring or early summer. We targeted a mix of solitary and social nests, where we sampled the solitary female or a group of 2–5 individuals from a multi-female colony, respectively. We recorded the locality of each nest using a portable GPS. At each population, we took care to sample from multiple clusters of colonies separated by several meters or more.

### Phenotyping

We assessed the social structure of each colony in the field by counting the number of adult and subadult females present in the nest. Females were deemed to exhibit the subsocial strategy if only a singleton reproductive female resided in the nest, whereas females were deemed to exhibit the social strategy if two or more reproductive females cohabitated within the same next. To evaluate where females fell along the bold vs. shy axis, we transported nests back to laboratory at the University of Tennessee, Knoxville for startle tests, which are a common assay for evaluating boldness in spiders [25, 31, 44, 52]. In brief, spiders were taken from their nests and placed within a square open field and given 30 s to acclimate. Following this acclimation period two rapid jets of air were administered to the anterior prosoma of the spider using an infant nose-cleaning bulb. This stimulus elicits a death feign response in *A. studiosus* where females draw their legs tightly against their body and remain in a huddled position for a variable amount of time. We recorded the latency for each female to emerge from the huddle and move one body length. Individuals that hold a huddle for a longer period are deemed to be shy, whereas individuals that emerge from huddles swiftly are deemed to be bold. Following the phenotypic assessment, we placed spiders in 2 mL tubes containing 95% ethanol and transported them to the molecular laboratory. Open fields were cleaned with 95% ethanol between behavioral assays.

### Sequencing and bioinformatics

For whole genome sequencing, we extracted DNA from the focal individual using a modified Insect Tissue protocol (based on Qiagen’s DNeasy protocol). In particular, we used alternative spin columns (BPI-tech.com) and eluted in 30 uL of AE buffer. We then sent the DNA to the University of Lausanne Genomics Core facility for library preparation with the TruSeq DNA kit and insert size of 500 bp and carried out 100 bp paired end sequencing on two Illumina 2500 lanes. We obtained 164 M unique reads or 33 Gbp, which amounted to approximately 14x coverage (the latter estimated through the kmercountexact.sh function in BBMap, [14]).

We assembled the genome using the following pipeline: we used a custom shell command to remove reads that failed the Illumina chastity filter, we removed PCR duplicates with FastUniq [55], we combined overlapping paired end reads and removed adapters with PEAR [56], and we trimmed low quality bases with Sickle [30]. With these processed sequences, we assembled reads using SOAPdenovo2 [36] with a range of k values from 29 to 61, as well as with the multi k function. Small k values tended to yield larger but more fragmented assemblies, and we selected k43 as the best compromise between contiguity and completeness. We then scaffolded this assembly with SSPACE [10] and filled gaps with GapFiller [11].

For linkage map and association study processing, we extracted DNA following the protocol described above. We then used a customized ddRADseq protocol to amplify restriction fragments distributed across the genome [12, 42]. Briefly, we digested the genomic DNA with SbfI and MseI enzymes, ligated unique barcoded adapters to the SbfI cut site, removed small DNA fragments from each individual using AMPure beads (Agencourt), carried out PCR in three parallel reactions, size-selected fragments in the 400–500 bp range through a gel extraction, and purified the resulting DNA using a gel extraction kit (Qiagen). Finally, we sequenced each library on two Illumina 2500 lanes at the University of Lausanne genomics core facility. For the linkage map library, we used 100 bp single end reads, while we used 100 bp paired end sequencing for the association study.

For each library, we demultiplexed the sequence data using the process_radtags function in Stacks [15], aligned RADtags to our draft genome using Bowtie2 [33], and called variants using Samtools [35]. We filtered the resulting markers in VCFtools [18]. For the linkage maps, we initially separated the two families and carried out filtering independently; we retained genotype calls with a quality score greater than 20 (‘mingq = 20’), we retained markers present in at least 80% of individuals (geno = 80), and we removed markers with a minor allele frequency less than 15% (maf = 0.15). We examined missing data by individual, and we ultimately retained 32 and 36 offspring that had high quality genotype calls for at least 50% of loci; with these smaller families, we redid the geno and maf filtering.

For the association study, we retained markers that had a minimum genotype quality score of 20 or more (mingq = 20), were missing in less than 80% (geno = 0.8) of individuals, and that exhibited a minor allele frequency over 5% (maf = 0.05). We then examined the level of missing data in individuals and we removed 16 samples for which 25% or more loci were missing. Moving forward with 210 individuals, we reran the filters described above and generated a list of 4179 high-quality markers that were present in the majority of samples (mean read depth per site = 114x). In total, the sociality association test included 210 individuals at 4179 loci; four additional individuals were excluded from the boldness assay due to early mortality, resulting in a comparison of 206 individuals at 4179 loci.

### Linkage map assembly

After filtering the data, we determined which markers were heterozygous in each maternal sample. Using VCFtools, we retained the list of maternally informative markers for each group of siblings for subsequent processing, in order to develop a maternal map. We then surveyed the sibling groups for markers that were variable among siblings, but homozygous in the mother, and retained these markers in order to develop a paternal map for each family. In order to construct the linkage maps, we exported the four datasets as 012 text files for manual conversion to MSTmap input format [54]. To account for the unknown phase of maternal and paternal genotypes, we duplicated each marker in the dataset, calling each genotype ‘A’ in one copy and ‘B’ in the other, following the procedure of Gadau [21]. We tested different parameter values in MSTmap. In particular, we tried cut_off_p_values ranging from 0.0005 to 0.000005. In general, larger *p*-value cut-offs resulted in too many markers being lumped together in single linkage groups, while small cut-offs resulted in greater fragmentation. For each map, we selected an intermediate p-value cut-off that provided a compromise between these extremes. We show the MSTmap parameter values in Additional file 1: Table S1. Before inferring male genotypes to construct paternal linkage maps, we inspected relationships among sib-groups. Based on principal component analyses (Fig. 1—1436 loci for P1 family, 7930 loci for P2 family) and assessment of relatedness (Additional file 1: Figure S2), we suspected that each female had mated with multiple males, though this contradicts a prior study that detected exclusive first-male sperm precedence in this species [28]. As a result, we could not construct paternal linkage maps for either family.

### Association study analysis

We imputed individual genotypes across all our initial ddRADseq loci using BEAGLE [13]. We then used the list of high-quality markers determined through the filtering process described in the previous section to retain only the best markers from our dataset, now with missing data added through imputation. We used PLINK [43] to generate files in the bim and fam formats needed to assess associations and correct for underlying population genetic structure in GEMMA [57]. For each trait (boldness and sociality), we added the phenotype scores manually to the fam file. We ran GEMMA to calculate the association between each response variable and all the polymorphic markers, including underlying population genetic structure as a random effect in the model. To do this, we first ran GEMMA on the imputed VCF files with the -gk flag to calculate the relatedness matrix (this was done separately for each trait due to missing data for boldness) and then with the -lmm flag to perform the mixed effects model. We identified statistical outliers using the Bonferroni correction to obtain a conservative alpha value (α = 3.38 × 10^− 5^); when statistical outliers were present, we compared the observed *p*-value distribution to the expected distribution by generating a quantile-quantile plot in R. We assessed the proportion of variance in boldness that was explained by the most significantly associated locus (Fig. 2) by comparing the sum of squares for genotype to the sum of squares total.

## Supplementary information

**Additional file 1.** Detailed information about linkage maps is shown, including a comparison between linkage maps for P1 family and P2 family (**Figure S1**), methods and results of linkage map family relatedness calculations (**Figure S2**), MSTmap parameters (**Table S1**), and full linkage map alignments and SNP positions for P1 family (**Table S2**) and P2 family (**Table S3**). In addition, the full list of individuals used in the association study along with their social and boldness phenotypes is provided (**Table S4**).

## Data Availability

The genetic data generated during this study are available in the NCBI GenBank repository BioProject PRJNA557053, which includes genome raw reads, association study RADseq reads, and linkage map RADseq reads. This Whole Genome Shotgun project has been deposited at DDBJ/ENA/GenBank under the accession VSFD00000000. The version described in this paper is version VSFD01000000. The phenotypic data are provided in the supplementary information file associated with this article.
