# Supplementary material for: Are personalities genetically determined? Inferences from subsocial spiders
Source: BMC Genomics. 2019 Nov 22;20:867. doi: 10.1186/s12864-019-6172-5 (PMC6873478; doi:10.1186/s12864-019-6172-5)
Supplement: Supplementary file 1 — Additional file 1. Detailed information about linkage maps is shown, including a comparison between linkage maps for P1 family and P2 family (Figure S1), methods and results of linkage map family relatedness calculations (Figure S2), MSTmap parameters (Table S1), and full linkage map alignments and SNP positions for P1 family (Table S2) and P2 family (Table S3). In addition, the full list of individuals used in the association study along with their social and boldness phenotypes is provided (Table S4). [file 12864_2019_6172_MOESM1_ESM.docx]

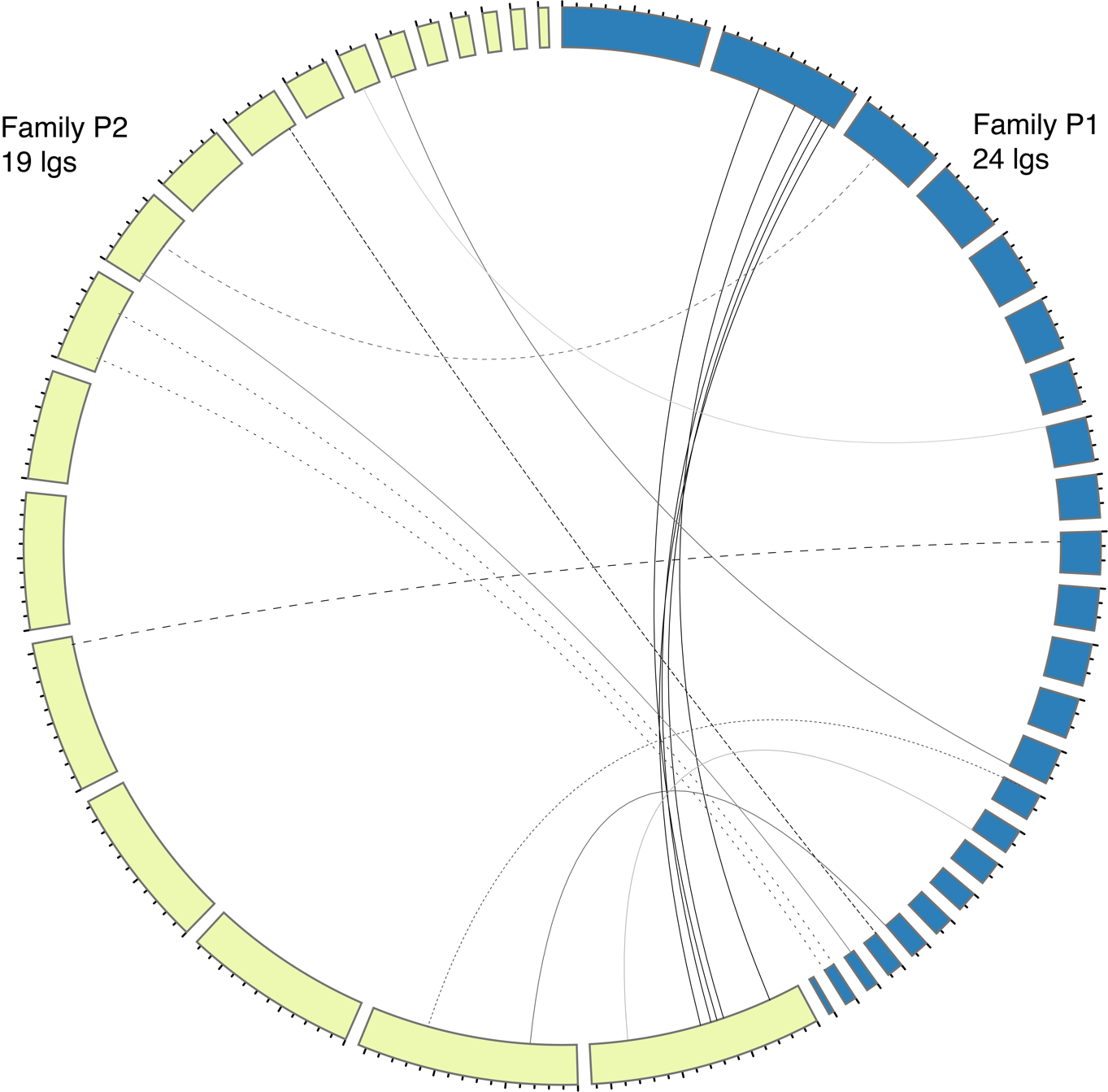
Supplementary materials for Purcell and Pruitt, **Are personalities genetically determined? Inferences from subsocial spiders**

**Figure S1.** We obtained high density linkage maps from two *A. studiosus* families that included mother and offspring. Maps represent recombination in the mother in both cases. Loci shared between the two maps are linked by connecting lines; lines connecting different P1 chromosomes are distinguished by different types of lines (e.g. some dotted, some dashed). Consecutive tick marks on the linkage map bars (blue for P1, yellow for P2) separate 20 cM.

**Relatedness calculations.** In order to verify our PCA-based inference that each sibling group used for linkage mapping contained one mother and two fathers (Fig. 1), we calculated pairwise relatedness. Relatedness is a relative measure of genetic similarity, and is biased downward when many individuals in a sample are close relatives. To mitigate this issue, we analyzed our linkage mapping families together with the spiders from our association study. We ran samtools mpileup on this full set of individuals (307 individuals), and then we filtered the data using VCFtools with the following parameters: minimum genotype quality score (minGQ) of 20, minimum read depth (minDP) of 1, missing in less than 80% of individuals (geno), and minimum minor allele count (mac) of 3. We then removed individual spiders with more than 50% missing data for a total dataset of 275 individuals and 6540 variant loci. We then used the VCFtools relatedness flag to calculate pairwise relatedness. For putative full siblings and parent-offspring relationships, the average relatedness was 0.2 (Fig. S2), which is still much lower than the expected average relatedness values of 0.5. We tested whether this discrepancy could be attributed to the fact that about 1/3 of the individuals in our full dataset were still members of two families, resulting in asymmetries within the dataset by further subsampling these families: we retained the two mothers and 3 offspring from each putative full sibling group, along with the remaining population sample. In this case, the average relatedness values for putative full sibs (across the four groups) was 0.503, for mother/offspring relationships (across two families) was 0.410, and for half sibs (across two families) was 0.273.

**Figure S2.** We calculated relatedness across our linkage mapping and association study sample (6540 SNPs, 275 individuals). Overall, we found that individuals from our putative full sib groups in families P1 and P2 showed the highest levels of relatedness (along with some nestmates from social groups). Relatedness of putative full sibling groups was in line with relatedness between mothers and presumed offspring in both families. We then compared putative half sib groups, and found a relatedness value that was about 50% of that exhibited by full sib groups, but still higher than the mean relatedness between non-nestmates from the same population (MH). We note that these relatedness values were much lower than the expected average relatedness of 0.5 for full siblings; we carried out additional analyses to determine the cause of this discrepancy (see Relatedness calculations section).

**Table S1**: Parameters used to generate linkage maps in MSTMap for spider families P1 and P2.

| **MSTmap Variable** | **P1 and P2** |
| --- | --- |
| Population_type | DH |
| Distance_function | Kosambi |
| Cut_off_p_value | 0.00005 |
| No_map_dist | 30 |
| No_map_size | 1 |
| Missing_threshold | 0.25, 0.15, respectively |
| Detect_bad_data | yes |
| Objective_function | ML |

**Table S2**: Maternal linkage map for P1 family.

| *A. Studiosus* scaffold | SNP position | Map position (cM) | Linkage Group |
| --- | --- | --- | --- |
| scaffold26904.1\|size12199 | 7623 | 0 | 1 |
| scaffold2559.1\|size26342 | 12702 | 8.192 | 1 |
| scaffold30083.1\|size14817 | 3956 | 23.364 | 1 |
| scaffold298560.1\|size2128 | 1538 | 35.998 | 1 |
| scaffold764362.1\|size488 | 161 | 43.897 | 1 |
| scaffold118021.1\|size5737 | 2342 | 56.091 | 1 |
| scaffold881817.1\|size231 | 58 | 73.537 | 1 |
| scaffold214788.1\|size3091 | 623 | 81.829 | 1 |
| scaffold349323.1\|size1697 | 50 | 87.194 | 1 |
| scaffold349323.1\|size1697 | 81 | 87.194 | 1 |
| scaffold349323.1\|size1697 | 96 | 87.194 | 1 |
| scaffold49433.1\|size9065 | 3578 | 93.437 | 1 |
| scaffold49433.1\|size9065 | 3649 | 93.437 | 1 |
| scaffold750.1\|size34993 | 22426 | 105.548 | 1 |
| scaffold918698.1\|size214 | 79 | 109.78 | 1 |
| scaffold36378.1\|size10608 | 7851 | 117.693 | 1 |
| scaffold330542.1\|size1842 | 143 | 127.386 | 1 |
| scaffold39179.1\|size10219 | 4051 | 143.629 | 1 |
| scaffold1477738.1\|size139 | 79 | 154.444 | 1 |
| scaffold68440.1\|size13101 | 8839 | 158.746 | 1 |
| scaffold68440.1\|size13101 | 8849 | 158.746 | 1 |
| scaffold191208.1\|size6895 | 3598 | 168.823 | 1 |
| scaffold49433.1\|size9065 | 3623 | 175.108 | 1 |
| scaffold312328.1\|size2044 | 238 | 199.657 | 1 |
| scaffold1708599.1\|size129 | 72 | 207.392 | 1 |
| scaffold82909.1\|size6939 | 4778 | 0 | 2 |
| scaffold10016.1\|size17787 | 6337 | 11.902 | 2 |
| scaffold82909.1\|size6939 | 4758 | 18.379 | 2 |
| scaffold2485.1\|size41557 | 32876 | 26.293 | 2 |
| scaffold2076.1\|size27669 | 21550 | 33.351 | 2 |
| scaffold76437.1\|size7546 | 4830 | 43.4 | 2 |
| scaffold68609.1\|size7526 | 2423 | 52.278 | 2 |
| scaffold81460.1\|size6769 | 3298 | 61.593 | 2 |
| scaffold45098.1\|size9514 | 7492 | 69.443 | 2 |
| scaffold68364.1\|size7543 | 1802 | 75.899 | 2 |
| scaffold15945.1\|size16674 | 9162 | 75.899 | 2 |
| scaffold15945.1\|size16674 | 9174 | 75.899 | 2 |
| scaffold15945.1\|size16674 | 9206 | 75.899 | 2 |
| scaffold15945.1\|size16674 | 9176 | 75.899 | 2 |
| scaffold60289.1\|size8127 | 1087 | 85.685 | 2 |
| scaffold1768270.1\|size125 | 55 | 93.363 | 2 |
| scaffold80804.1\|size12416 | 669 | 102.223 | 2 |
| scaffold80804.1\|size12416 | 733 | 102.223 | 2 |
| scaffold171884.1\|size4810 | 3442 | 102.223 | 2 |
| scaffold1066084.1\|size177 | 121 | 107.098 | 2 |
| scaffold20306.1\|size13747 | 11486 | 113.408 | 2 |
| scaffold20306.1\|size13747 | 11538 | 113.408 | 2 |
| scaffold112960.1\|size5402 | 1022 | 131.132 | 2 |
| scaffold112960.1\|size5402 | 1047 | 131.132 | 2 |
| scaffold112960.1\|size5402 | 1081 | 131.132 | 2 |
| scaffold11364.1\|size17050 | 1918 | 131.132 | 2 |
| scaffold12041.1\|size16713 | 1001 | 135.143 | 2 |
| scaffold60955.1\|size8074 | 4376 | 146.29 | 2 |
| scaffold87292.1\|size6718 | 835 | 156.933 | 2 |
| scaffold87292.1\|size6718 | 836 | 156.933 | 2 |
| scaffold1116.1\|size33099 | 22288 | 169.961 | 2 |
| scaffold1116.1\|size33099 | 22323 | 169.961 | 2 |
| scaffold1809119.1\|size123 | 72 | 180.569 | 2 |
| scaffold4912.1\|size22140 | 13704 | 193.499 | 2 |
| scaffold254305.1\|size2583 | 620 | 203.265 | 2 |
| scaffold254305.1\|size2583 | 622 | 203.265 | 2 |
| scaffold1750987.1\|size127 | 33 | 0 | 3 |
| scaffold1750987.1\|size127 | 44 | 0 | 3 |
| scaffold1750987.1\|size127 | 71 | 0 | 3 |
| scaffold1750987.1\|size127 | 84 | 0 | 3 |
| scaffold1615525.1\|size133 | 44 | 12.537 | 3 |
| scaffold1615525.1\|size133 | 92 | 12.537 | 3 |
| scaffold164388.1\|size3993 | 3074 | 34.264 | 3 |
| scaffold164388.1\|size3993 | 3080 | 41.454 | 3 |
| scaffold164388.1\|size3993 | 3086 | 41.454 | 3 |
| scaffold401305.1\|size1415 | 1151 | 58.244 | 3 |
| scaffold98287.1\|size5970 | 337 | 75.005 | 3 |
| scaffold1585588.1\|size134 | 45 | 92.918 | 3 |
| scaffold1585588.1\|size134 | 72 | 92.918 | 3 |
| scaffold2141102.1\|size107 | 44 | 108.668 | 3 |
| scaffold2141102.1\|size107 | 101 | 123.093 | 3 |
| scaffold146978.1\|size4397 | 1454 | 0 | 4 |
| scaffold146978.1\|size4397 | 1475 | 0 | 4 |
| scaffold146978.1\|size4397 | 1523 | 0 | 4 |
| scaffold146978.1\|size4397 | 1524 | 0 | 4 |
| scaffold142480.1\|size4511 | 1581 | 12.28 | 4 |
| scaffold67285.1\|size8344 | 6049 | 23.514 | 4 |
| scaffold4038.1\|size23446 | 15080 | 30.06 | 4 |
| scaffold925.1\|size33000 | 19910 | 35.59 | 4 |
| scaffold2753.1\|size25849 | 15972 | 35.59 | 4 |
| scaffold9767.1\|size17951 | 11256 | 42.741 | 4 |
| scaffold330331.1\|size1844 | 1068 | 55.271 | 4 |
| scaffold134118.1\|size5377 | 2601 | 66.593 | 4 |
| scaffold134118.1\|size5377 | 2605 | 66.593 | 4 |
| scaffold134118.1\|size5377 | 2602 | 81.752 | 4 |
| scaffold29749.1\|size11950 | 4916 | 90.955 | 4 |
| scaffold29749.1\|size11950 | 4870 | 90.955 | 4 |
| scaffold2125323.1\|size108 | 79 | 102.739 | 4 |
| scaffold22102.1\|size13275 | 5078 | 0 | 5 |
| scaffold414450.1\|size1277 | 81 | 11.208 | 5 |
| scaffold18082.1\|size14617 | 4436 | 20.154 | 5 |
| scaffold191002.1\|size3481 | 2452 | 37.903 | 5 |
| scaffold380672.1\|size1577 | 246 | 45.549 | 5 |
| scaffold25654.1\|size14148 | 2177 | 50.483 | 5 |
| scaffold25654.1\|size14148 | 2223 | 50.483 | 5 |
| scaffold16966.1\|size14787 | 6284 | 55.889 | 5 |
| scaffold4112.1\|size23314 | 4659 | 64.787 | 5 |
| scaffold4112.1\|size23314 | 4680 | 64.787 | 5 |
| scaffold4112.1\|size23314 | 4751 | 72.349 | 5 |
| scaffold4112.1\|size23314 | 4779 | 72.349 | 5 |
| scaffold127564.1\|size4932 | 3348 | 78.624 | 5 |
| scaffold127564.1\|size4932 | 3349 | 78.624 | 5 |
| scaffold51136.1\|size8901 | 4910 | 84.795 | 5 |
| scaffold84411.1\|size6616 | 1355 | 84.795 | 5 |
| scaffold84411.1\|size6616 | 1356 | 84.795 | 5 |
| scaffold29400.1\|size11724 | 1360 | 0 | 6 |
| scaffold41792.1\|size11767 | 5919 | 8.236 | 6 |
| scaffold108983.1\|size5544 | 3182 | 25.065 | 6 |
| scaffold105413.1\|size5680 | 649 | 34.209 | 6 |
| scaffold43109.1\|size9737 | 6442 | 43.305 | 6 |
| scaffold105413.1\|size5680 | 522 | 49.885 | 6 |
| scaffold105413.1\|size5680 | 540 | 49.885 | 6 |
| scaffold108516.1\|size5561 | 491 | 49.885 | 6 |
| scaffold86290.1\|size13138 | 5118 | 56.554 | 6 |
| scaffold162310.1\|size4038 | 69 | 66.571 | 6 |
| scaffold162310.1\|size4038 | 74 | 66.571 | 6 |
| scaffold162310.1\|size4038 | 80 | 66.571 | 6 |
| scaffold429516.1\|size2654 | 1296 | 73.826 | 6 |
| scaffold83753.1\|size6648 | 2767 | 0 | 7 |
| scaffold209912.1\|size3154 | 509 | 2.854 | 7 |
| scaffold36467.1\|size10596 | 6199 | 15.59 | 7 |
| scaffold52265.1\|size8794 | 833 | 22.889 | 7 |
| scaffold52265.1\|size8794 | 838 | 22.889 | 7 |
| scaffold52265.1\|size8794 | 850 | 22.889 | 7 |
| scaffold24704.1\|size12664 | 9208 | 22.889 | 7 |
| scaffold64450.1\|size7817 | 2088 | 22.889 | 7 |
| scaffold64450.1\|size7817 | 2093 | 22.889 | 7 |
| scaffold98897.1\|size5945 | 320 | 31.483 | 7 |
| scaffold20204.1\|size13778 | 7997 | 39.4 | 7 |
| scaffold104925.1\|size5700 | 4708 | 48.084 | 7 |
| scaffold611805.1\|size662 | 390 | 62.466 | 7 |
| scaffold81948.1\|size6742 | 4633 | 0 | 8 |
| scaffold2229656.1\|size104 | 77 | 2.278 | 8 |
| scaffold15825.1\|size15162 | 4308 | 9.98 | 8 |
| scaffold15825.1\|size15162 | 4309 | 9.98 | 8 |
| scaffold1658.1\|size29537 | 2673 | 25.268 | 8 |
| scaffold419854.1\|size1250 | 460 | 36.441 | 8 |
| scaffold14181.1\|size15781 | 3195 | 45.334 | 8 |
| scaffold58699.1\|size8416 | 2795 | 55.866 | 8 |
| scaffold152558.1\|size4777 | 1216 | 61.253 | 8 |
| scaffold311667.1\|size2048 | 379 | 0 | 9 |
| scaffold311667.1\|size2048 | 397 | 0 | 9 |
| scaffold311667.1\|size2048 | 444 | 0 | 9 |
| scaffold422423.1\|size1239 | 374 | 12.155 | 9 |
| scaffold2132991.1\|size108 | 63 | 12.155 | 9 |
| scaffold933139.1\|size208 | 161 | 22.397 | 9 |
| scaffold7911.1\|size19198 | 12267 | 30.257 | 9 |
| scaffold54350.1\|size8614 | 4323 | 36.343 | 9 |
| scaffold5856.1\|size21063 | 16315 | 47.606 | 9 |
| scaffold5856.1\|size21063 | 16322 | 47.606 | 9 |
| scaffold5856.1\|size21063 | 16375 | 47.606 | 9 |
| scaffold52642.1\|size8761 | 7542 | 59.922 | 9 |
| scaffold240854.1\|size2716 | 1953 | 0 | 10 |
| scaffold240854.1\|size2716 | 1954 | 0 | 10 |
| scaffold6326.1\|size20589 | 15126 | 13.494 | 10 |
| scaffold4142.1\|size23275 | 3828 | 32.919 | 10 |
| scaffold40775.1\|size10019 | 7875 | 32.919 | 10 |
| scaffold561063.1\|size708 | 596 | 44.201 | 10 |
| scaffold40976.1\|size13645 | 9187 | 59.588 | 10 |
| scaffold40976.1\|size13645 | 9233 | 59.588 | 10 |
| scaffold40976.1\|size13645 | 9235 | 59.588 | 10 |
| scaffold60055.1\|size9397 | 6855 | 59.588 | 10 |
| scaffold60055.1\|size9397 | 6859 | 59.588 | 10 |
| scaffold60055.1\|size9397 | 6860 | 59.588 | 10 |
| scaffold1960667.1\|size115 | 40 | 0 | 11 |
| scaffold117872.1\|size5230 | 2750 | 8.631 | 11 |
| scaffold136027.1\|size4679 | 1004 | 18.804 | 11 |
| scaffold59442.1\|size8191 | 3392 | 41.021 | 11 |
| scaffold59442.1\|size8191 | 3413 | 41.021 | 11 |
| scaffold59442.1\|size8191 | 3463 | 41.021 | 11 |
| scaffold325873.1\|size11487 | 3993 | 50.429 | 11 |
| scaffold117872.1\|size5230 | 2843 | 59.032 | 11 |
| scaffold528624.1\|size1503 | 664 | 0 | 12 |
| scaffold528624.1\|size1503 | 663 | 0 | 12 |
| scaffold805010.1\|size307 | 48 | 16.96 | 12 |
| scaffold805010.1\|size307 | 87 | 16.96 | 12 |
| scaffold330331.1\|size1844 | 991 | 40.491 | 12 |
| scaffold306080.1\|size2083 | 626 | 44.813 | 12 |
| scaffold287193.1\|size2204 | 284 | 49.763 | 12 |
| scaffold308160.1\|size2071 | 468 | 54.188 | 12 |
| scaffold287193.1\|size2204 | 237 | 58.644 | 12 |
| scaffold287193.1\|size2204 | 276 | 58.644 | 12 |
| scaffold287193.1\|size2204 | 282 | 58.644 | 12 |
| scaffold287193.1\|size2204 | 297 | 58.644 | 12 |
| scaffold14530.1\|size16706 | 3348 | 0 | 13 |
| scaffold478666.1\|size1115 | 433 | 9.372 | 13 |
| scaffold395190.1\|size1496 | 1398 | 13.851 | 13 |
| scaffold307372.1\|size2075 | 797 | 18.243 | 13 |
| scaffold10562.1\|size26655 | 4642 | 35.103 | 13 |
| scaffold16535.1\|size14926 | 10569 | 42.679 | 13 |
| scaffold52223.1\|size8798 | 5261 | 42.679 | 13 |
| scaffold52223.1\|size8798 | 5262 | 42.679 | 13 |
| scaffold20876.1\|size15172 | 6686 | 56.629 | 13 |
| scaffold20876.1\|size15172 | 6728 | 56.629 | 13 |
| scaffold146768.1\|size4403 | 1127 | 0 | 14 |
| scaffold1512.1\|size30096 | 18999 | 11.713 | 14 |
| scaffold8707.1\|size18635 | 10973 | 20.074 | 14 |
| scaffold135235.1\|size4702 | 2263 | 26.221 | 14 |
| scaffold349572.1\|size1696 | 51 | 32.136 | 14 |
| scaffold14590.1\|size15642 | 14306 | 42.935 | 14 |
| scaffold2340779.1\|size101 | 80 | 42.935 | 14 |
| scaffold580841.1\|size686 | 452 | 49.404 | 14 |
| scaffold429709.1\|size1214 | 853 | 0 | 15 |
| scaffold429709.1\|size1214 | 854 | 0 | 15 |
| scaffold429709.1\|size1214 | 889 | 0 | 15 |
| scaffold2202153.1\|size105 | 44 | 8.524 | 15 |
| scaffold2202153.1\|size105 | 62 | 8.524 | 15 |
| scaffold172243.1\|size3828 | 1487 | 16.646 | 15 |
| scaffold9386.1\|size18184 | 10969 | 20.221 | 15 |
| scaffold240817.1\|size3224 | 504 | 23.199 | 15 |
| scaffold240817.1\|size3224 | 512 | 23.199 | 15 |
| scaffold50900.1\|size8926 | 483 | 27.45 | 15 |
| scaffold59023.1\|size8492 | 3544 | 37.062 | 15 |
| scaffold124584.1\|size5017 | 1334 | 37.062 | 15 |
| scaffold109197.1\|size5537 | 3087 | 0 | 16 |
| scaffold81506.1\|size6766 | 305 | 12.789 | 16 |
| scaffold81506.1\|size6766 | 346 | 12.789 | 16 |
| scaffold90993.1\|size8333 | 6478 | 24.649 | 16 |
| scaffold1207.1\|size31162 | 9182 | 31.884 | 16 |
| scaffold73238.1\|size16878 | 8370 | 35.078 | 16 |
| scaffold83017.1\|size6686 | 4191 | 35.078 | 16 |
| scaffold125816.1\|size4982 | 366 | 35.078 | 16 |
| scaffold149653.1\|size4328 | 2937 | 35.078 | 16 |
| scaffold94117.1\|size6146 | 4357 | 35.078 | 16 |
| scaffold313256.1\|size2037 | 1260 | 0 | 17 |
| scaffold52108.1\|size8810 | 6417 | 5.266 | 17 |
| scaffold69670.1\|size7453 | 3927 | 11.886 | 17 |
| scaffold82060.1\|size6737 | 3633 | 17.093 | 17 |
| scaffold73200.1\|size7236 | 6150 | 22.867 | 17 |
| scaffold643.1\|size35624 | 22815 | 34.117 | 17 |
| scaffold126772.1\|size5207 | 2361 | 0 | 18 |
| scaffold126772.1\|size5207 | 2392 | 0 | 18 |
| scaffold130441.1\|size5929 | 4021 | 11.954 | 18 |
| scaffold58475.1\|size8795 | 4258 | 16.493 | 18 |
| scaffold52178.1\|size8802 | 5771 | 28.743 | 18 |
| scaffold52178.1\|size8802 | 5718 | 28.743 | 18 |
| scaffold52178.1\|size8802 | 5750 | 28.743 | 18 |
| scaffold379109.1\|size1583 | 297 | 0 | 19 |
| scaffold115565.1\|size5310 | 2731 | 1.897 | 19 |
| scaffold13539.1\|size16050 | 2928 | 6.998 | 19 |
| scaffold287035.1\|size2206 | 1379 | 14.899 | 19 |
| scaffold52998.1\|size8728 | 334 | 24.17 | 19 |
| scaffold546691.1\|size1029 | 615 | 27.401 | 19 |
| scaffold1848552.1\|size121 | 33 | 0 | 20 |
| scaffold1848552.1\|size121 | 69 | 0 | 20 |
| scaffold92262.1\|size7861 | 3409 | 13.219 | 20 |
| scaffold92262.1\|size7861 | 3428 | 13.219 | 20 |
| scaffold196723.1\|size3524 | 663 | 23.912 | 20 |
| scaffold46470.1\|size9371 | 757 | 26.505 | 20 |
| scaffold46470.1\|size9371 | 758 | 26.505 | 20 |
| scaffold46470.1\|size9371 | 760 | 26.505 | 20 |
| scaffold148684.1\|size4352 | 2841 | 0 | 21 |
| scaffold148684.1\|size4352 | 2844 | 0 | 21 |
| scaffold148684.1\|size4352 | 2862 | 0 | 21 |
| scaffold148684.1\|size4352 | 2892 | 0 | 21 |
| scaffold223338.1\|size2983 | 2025 | 8.587 | 21 |
| scaffold223338.1\|size2983 | 2040 | 8.587 | 21 |
| scaffold3.1\|size78572 | 42382 | 13.067 | 21 |
| scaffold1821390.1\|size122 | 63 | 19.399 | 21 |
| scaffold1821390.1\|size122 | 67 | 19.399 | 21 |
| scaffold1821390.1\|size122 | 79 | 19.399 | 21 |
| scaffold1821390.1\|size122 | 91 | 19.399 | 21 |
| scaffold192235.1\|size3461 | 1037 | 22.938 | 21 |
| scaffold188022.1\|size3531 | 300 | 0 | 22 |
| scaffold1977.1\|size28740 | 8168 | 4.394 | 22 |
| scaffold75092.1\|size14164 | 1829 | 14.412 | 22 |
| scaffold75092.1\|size14164 | 1894 | 14.412 | 22 |
| scaffold417170.1\|size1615 | 1085 | 15.11 | 22 |
| scaffold16885.1\|size14811 | 361 | 16.897 | 22 |
| scaffold226991.1\|size3847 | 573 | 0 | 23 |
| scaffold226991.1\|size3847 | 592 | 0 | 23 |
| scaffold68162.1\|size7558 | 1803 | 16.166 | 23 |
| scaffold68162.1\|size7558 | 1810 | 16.166 | 23 |
| scaffold68162.1\|size7558 | 1821 | 16.166 | 23 |
| scaffold217115.1\|size3063 | 1742 | 0 | 24 |
| scaffold217115.1\|size3063 | 1745 | 0 | 24 |
| scaffold141420.1\|size6134 | 367 | 5.141 | 24 |
| scaffold37232.1\|size10485 | 930 | 7.487 | 24 |
| scaffold122627.1\|size5075 | 1553 | 7.487 | 24 |

**Table S3**: Maternal linkage map for P2 family.

| *A. studiosus* scaffold | SNP position | Map position (cM) | Linkage group |
| --- | --- | --- | --- |
| scaffold239431.1\|size2735 | 788 | 0 | 1 |
| scaffold8874.1\|size18532 | 14259 | 11.735 | 1 |
| scaffold8874.1\|size18532 | 14302 | 11.735 | 1 |
| scaffold32740.1\|size11155 | 1023 | 18.902 | 1 |
| scaffold500153.1\|size1069 | 885 | 22.362 | 1 |
| scaffold68578.1\|size7527 | 1060 | 22.362 | 1 |
| scaffold386271.1\|size1553 | 749 | 27.894 | 1 |
| scaffold42064.1\|size10043 | 1340 | 27.894 | 1 |
| scaffold4728.1\|size22391 | 8656 | 32.873 | 1 |
| scaffold174613.1\|size3773 | 2095 | 35.609 | 1 |
| scaffold142409.1\|size4513 | 1254 | 43 | 1 |
| scaffold12041.1\|size16713 | 1001 | 47.952 | 1 |
| scaffold45132.1\|size9511 | 4925 | 58.07 | 1 |
| scaffold149444.1\|size4333 | 3014 | 66.322 | 1 |
| scaffold34540.1\|size10880 | 5651 | 66.322 | 1 |
| scaffold30735.1\|size11489 | 5671 | 72.971 | 1 |
| scaffold11462.1\|size16989 | 7998 | 83.33 | 1 |
| scaffold855959.1\|size510 | 112 | 91.165 | 1 |
| scaffold855959.1\|size510 | 74 | 91.165 | 1 |
| scaffold855959.1\|size510 | 75 | 91.165 | 1 |
| scaffold59858.1\|size9244 | 809 | 102.218 | 1 |
| scaffold59858.1\|size9244 | 815 | 102.218 | 1 |
| scaffold113035.1\|size5399 | 1187 | 113.26 | 1 |
| scaffold72749.1\|size7263 | 5799 | 122.381 | 1 |
| scaffold1809119.1\|size123 | 72 | 122.381 | 1 |
| scaffold4157.1\|size23247 | 16305 | 127.696 | 1 |
| scaffold15945.1\|size16674 | 9176 | 132.505 | 1 |
| scaffold1116.1\|size33099 | 22288 | 142.109 | 1 |
| scaffold1116.1\|size33099 | 22323 | 142.109 | 1 |
| scaffold23520.1\|size17239 | 1016 | 150.695 | 1 |
| scaffold20041.1\|size23869 | 14829 | 158.366 | 1 |
| scaffold4912.1\|size22140 | 13555 | 158.366 | 1 |
| scaffold443057.1\|size1180 | 83 | 169.321 | 1 |
| scaffold260214.1\|size2528 | 697 | 176.955 | 1 |
| scaffold5654.1\|size21273 | 473 | 182.983 | 1 |
| scaffold11561.1\|size16936 | 1271 | 190.419 | 1 |
| scaffold176601.1\|size8378 | 6792 | 203.744 | 1 |
| scaffold155651.1\|size4179 | 2486 | 211.521 | 1 |
| scaffold155651.1\|size4179 | 2487 | 211.521 | 1 |
| scaffold155651.1\|size4179 | 2495 | 211.521 | 1 |
| scaffold155651.1\|size4179 | 2556 | 211.521 | 1 |
| scaffold24819.1\|size12638 | 1547 | 211.521 | 1 |
| scaffold11011.1\|size17224 | 9252 | 219.46 | 1 |
| scaffold884506.1\|size478 | 327 | 224.013 | 1 |
| scaffold175788.1\|size3748 | 1783 | 229.492 | 1 |
| scaffold24731.1\|size16212 | 2474 | 236.931 | 1 |
| scaffold35989.1\|size10899 | 5291 | 239.824 | 1 |
| scaffold116775.1\|size5856 | 3930 | 251.48 | 1 |
| scaffold58461.1\|size8269 | 3797 | 262.322 | 1 |
| scaffold58461.1\|size8269 | 3801 | 262.322 | 1 |
| scaffold1029.1\|size32294 | 9304 | 262.322 | 1 |
| scaffold358380.1\|size1659 | 1336 | 262.322 | 1 |
| scaffold990999.1\|size450 | 253 | 262.322 | 1 |
| scaffold384623.1\|size1560 | 332 | 266.264 | 1 |
| scaffold73238.1\|size16878 | 8370 | 270.284 | 1 |
| scaffold73238.1\|size16878 | 8331 | 280.185 | 1 |
| scaffold73238.1\|size16878 | 8394 | 280.185 | 1 |
| scaffold1500141.1\|size138 | 86 | 296.475 | 1 |
| scaffold54361.1\|size8613 | 309 | 300.288 | 1 |
| scaffold30872.1\|size11783 | 1011 | 304.908 | 1 |
| scaffold725049.1\|size597 | 311 | 308.042 | 1 |
| scaffold761172.1\|size950 | 284 | 308.042 | 1 |
| scaffold725049.1\|size597 | 363 | 308.042 | 1 |
| scaffold51114.1\|size8904 | 1427 | 314.117 | 1 |
| scaffold51114.1\|size8904 | 1448 | 314.117 | 1 |
| scaffold214755.1\|size3091 | 217 | 314.117 | 1 |
| scaffold569982.1\|size1094 | 896 | 314.117 | 1 |
| scaffold569982.1\|size1094 | 925 | 314.117 | 1 |
| scaffold777227.1\|size967 | 519 | 314.117 | 1 |
| scaffold452908.1\|size1452 | 1471 | 326.664 | 1 |
| scaffold452908.1\|size1452 | 1503 | 326.664 | 1 |
| scaffold20669.1\|size13650 | 3301 | 0 | 2 |
| scaffold196625.1\|size4881 | 1095 | 10.398 | 2 |
| scaffold196625.1\|size4881 | 1122 | 10.398 | 2 |
| scaffold2273305.1\|size103 | 60 | 16.663 | 2 |
| scaffold100736.1\|size6528 | 4897 | 23.628 | 2 |
| scaffold353166.1\|size3830 | 2650 | 31.313 | 2 |
| scaffold353166.1\|size3830 | 2684 | 31.313 | 2 |
| scaffold61296.1\|size8047 | 3425 | 38.472 | 2 |
| scaffold157400.1\|size4424 | 124 | 45.977 | 2 |
| scaffold95790.1\|size6078 | 2323 | 55.644 | 2 |
| scaffold162226.1\|size4040 | 3649 | 55.644 | 2 |
| scaffold21854.1\|size13340 | 1513 | 61.88 | 2 |
| scaffold121985.1\|size5096 | 1471 | 61.88 | 2 |
| scaffold46470.1\|size9371 | 757 | 68.728 | 2 |
| scaffold46470.1\|size9371 | 758 | 68.728 | 2 |
| scaffold591932.1\|size1268 | 603 | 68.728 | 2 |
| scaffold591932.1\|size1268 | 618 | 68.728 | 2 |
| scaffold21035.1\|size13551 | 8556 | 68.728 | 2 |
| scaffold29787.1\|size12001 | 2737 | 68.728 | 2 |
| scaffold46470.1\|size9371 | 760 | 72.957 | 2 |
| scaffold108121.1\|size6460 | 4541 | 83.007 | 2 |
| scaffold108121.1\|size6460 | 4521 | 83.007 | 2 |
| scaffold50782.1\|size8936 | 1149 | 99.461 | 2 |
| scaffold759923.1\|size1255 | 1493 | 114.568 | 2 |
| scaffold759923.1\|size1255 | 1528 | 114.568 | 2 |
| scaffold45148.1\|size9508 | 7800 | 130.065 | 2 |
| scaffold210943.1\|size3140 | 354 | 138.721 | 2 |
| scaffold210943.1\|size3140 | 401 | 147.003 | 2 |
| scaffold4668.1\|size31401 | 11310 | 159.162 | 2 |
| scaffold31918.1\|size11292 | 2158 | 159.162 | 2 |
| scaffold36057.1\|size10654 | 2744 | 159.162 | 2 |
| scaffold1054345.1\|size179 | 139 | 159.162 | 2 |
| scaffold45201.1\|size9503 | 44 | 159.162 | 2 |
| scaffold269234.1\|size2960 | 2713 | 159.162 | 2 |
| scaffold872155.1\|size236 | 65 | 159.162 | 2 |
| scaffold58897.1\|size8234 | 5109 | 163.475 | 2 |
| scaffold72529.1\|size9967 | 671 | 166.751 | 2 |
| scaffold262227.1\|size2508 | 416 | 173.013 | 2 |
| scaffold109176.1\|size5740 | 4494 | 175.527 | 2 |
| scaffold387073.1\|size1549 | 1244 | 180.728 | 2 |
| scaffold387073.1\|size1549 | 1264 | 180.728 | 2 |
| scaffold12365.1\|size16571 | 3162 | 189.048 | 2 |
| scaffold238818.1\|size2743 | 405 | 192.737 | 2 |
| scaffold238818.1\|size2743 | 440 | 192.737 | 2 |
| scaffold100901.1\|size6135 | 3793 | 192.737 | 2 |
| scaffold83398.1\|size6667 | 421 | 196.665 | 2 |
| scaffold38606.1\|size10506 | 8422 | 199.804 | 2 |
| scaffold89513.1\|size6362 | 6650 | 205.359 | 2 |
| scaffold22587.1\|size13153 | 8661 | 205.359 | 2 |
| scaffold369082.1\|size1619 | 775 | 205.359 | 2 |
| scaffold148189.1\|size4366 | 3684 | 213.861 | 2 |
| scaffold110749.1\|size5481 | 4495 | 221.365 | 2 |
| scaffold59023.1\|size8492 | 3608 | 226.5 | 2 |
| scaffold70160.1\|size7422 | 3247 | 234.796 | 2 |
| scaffold320114.1\|size2815 | 483 | 244.385 | 2 |
| scaffold24680.1\|size13451 | 9016 | 251.062 | 2 |
| scaffold872155.1\|size236 | 66 | 263.346 | 2 |
| scaffold22047.1\|size13285 | 9802 | 272.025 | 2 |
| scaffold59280.1\|size8204 | 98 | 278.201 | 2 |
| scaffold59280.1\|size8204 | 121 | 278.201 | 2 |
| scaffold159739.1\|size4091 | 766 | 286.603 | 2 |
| scaffold159739.1\|size4091 | 815 | 286.603 | 2 |
| scaffold617634.1\|size1497 | 450 | 292.238 | 2 |
| scaffold916844.1\|size214 | 115 | 298.667 | 2 |
| scaffold280347.1\|size2269 | 1195 | 304.441 | 2 |
| scaffold135664.1\|size4689 | 2170 | 307.851 | 2 |
| scaffold686.1\|size35231 | 25764 | 311.428 | 2 |
| scaffold686.1\|size35231 | 25806 | 311.428 | 2 |
| scaffold59280.1\|size8204 | 125 | 311.428 | 2 |
| scaffold280347.1\|size2269 | 1163 | 311.428 | 2 |
| scaffold617225.1\|size658 | 104 | 0 | 3 |
| scaffold40404.1\|size15896 | 12560 | 12.78 | 3 |
| scaffold223469.1\|size2981 | 1963 | 24.256 | 3 |
| scaffold272230.1\|size2378 | 2101 | 28.531 | 3 |
| scaffold22023.1\|size13293 | 8493 | 39.329 | 3 |
| scaffold68033.1\|size7566 | 683 | 47.088 | 3 |
| scaffold10566.1\|size17667 | 10120 | 52.774 | 3 |
| scaffold331720.1\|size1827 | 1487 | 64.953 | 3 |
| scaffold331720.1\|size1827 | 1470 | 64.953 | 3 |
| scaffold26426.1\|size12292 | 8927 | 87.018 | 3 |
| scaffold34761.1\|size11217 | 6362 | 97.397 | 3 |
| scaffold73070.1\|size7243 | 4184 | 101.648 | 3 |
| scaffold172350.1\|size3825 | 509 | 107.5 | 3 |
| scaffold172350.1\|size3825 | 530 | 107.5 | 3 |
| scaffold260712.1\|size2523 | 1285 | 119.431 | 3 |
| scaffold105335.1\|size5912 | 1739 | 119.431 | 3 |
| scaffold253652.1\|size2837 | 1057 | 130.162 | 3 |
| scaffold271824.1\|size2384 | 98 | 138.521 | 3 |
| scaffold83012.1\|size6686 | 2849 | 146.076 | 3 |
| scaffold123909.1\|size10428 | 1395 | 156.515 | 3 |
| scaffold24908.1\|size13399 | 6942 | 163.523 | 3 |
| scaffold164450.1\|size3993 | 840 | 163.523 | 3 |
| scaffold164450.1\|size3993 | 858 | 163.523 | 3 |
| scaffold34976.1\|size10816 | 8194 | 166.282 | 3 |
| scaffold106997.1\|size5617 | 103 | 169.912 | 3 |
| scaffold6651.1\|size20283 | 16870 | 174.347 | 3 |
| scaffold90190.1\|size6329 | 4186 | 174.347 | 3 |
| scaffold6338.1\|size31460 | 13012 | 178.931 | 3 |
| scaffold99394.1\|size5924 | 4321 | 178.931 | 3 |
| scaffold263114.1\|size2498 | 519 | 182.179 | 3 |
| scaffold14829.1\|size15556 | 9582 | 185.819 | 3 |
| scaffold165368.1\|size3973 | 1350 | 185.819 | 3 |
| scaffold22431.1\|size13192 | 11349 | 185.819 | 3 |
| scaffold273725.1\|size2355 | 1179 | 188.471 | 3 |
| scaffold18527.1\|size14265 | 3839 | 195.085 | 3 |
| scaffold103526.1\|size5755 | 602 | 201.527 | 3 |
| scaffold189965.1\|size3498 | 2839 | 212.628 | 3 |
| scaffold189965.1\|size3498 | 2884 | 212.628 | 3 |
| scaffold163765.1\|size4006 | 2009 | 219.84 | 3 |
| scaffold114371.1\|size5352 | 2669 | 226.33 | 3 |
| scaffold213495.1\|size3107 | 473 | 230.478 | 3 |
| scaffold14.1\|size67278 | 33112 | 234.976 | 3 |
| scaffold11713.1\|size16863 | 8996 | 240.032 | 3 |
| scaffold66232.1\|size7688 | 321 | 240.032 | 3 |
| scaffold97235.1\|size6158 | 1159 | 240.032 | 3 |
| scaffold242498.1\|size2698 | 1792 | 240.032 | 3 |
| scaffold79481.1\|size6874 | 2229 | 247.794 | 3 |
| scaffold420826.1\|size1245 | 505 | 0 | 4 |
| scaffold3263.1\|size24812 | 19849 | 16.103 | 4 |
| scaffold85171.1\|size6576 | 3643 | 40.557 | 4 |
| scaffold85171.1\|size6576 | 3645 | 40.557 | 4 |
| scaffold26363.1\|size12305 | 504 | 51.252 | 4 |
| scaffold44139.1\|size9622 | 3476 | 51.252 | 4 |
| scaffold326994.1\|size1890 | 241 | 56.985 | 4 |
| scaffold6161.1\|size36688 | 12567 | 59.406 | 4 |
| scaffold71292.1\|size8162 | 2527 | 59.406 | 4 |
| scaffold89165.1\|size6379 | 4574 | 65.021 | 4 |
| scaffold171967.1\|size3833 | 3169 | 65.021 | 4 |
| scaffold23047.1\|size15044 | 4101 | 70.935 | 4 |
| scaffold23047.1\|size15044 | 4150 | 70.935 | 4 |
| scaffold148109.1\|size4368 | 623 | 76.77 | 4 |
| scaffold127103.1\|size4945 | 109 | 76.77 | 4 |
| scaffold127103.1\|size4945 | 148 | 76.77 | 4 |
| scaffold24306.1\|size12748 | 6450 | 80.964 | 4 |
| scaffold7913.1\|size28411 | 22127 | 91.271 | 4 |
| scaffold53148.1\|size8715 | 448 | 91.271 | 4 |
| scaffold67972.1\|size12791 | 3691 | 93.855 | 4 |
| scaffold5840.1\|size21083 | 12008 | 98.927 | 4 |
| scaffold1971166.1\|size115 | 53 | 101.741 | 4 |
| scaffold17051.1\|size21501 | 13461 | 105.345 | 4 |
| scaffold6321.1\|size25669 | 18262 | 105.345 | 4 |
| scaffold6321.1\|size25669 | 18295 | 105.345 | 4 |
| scaffold38656.1\|size19473 | 10006 | 105.345 | 4 |
| scaffold38656.1\|size19473 | 10040 | 105.345 | 4 |
| scaffold56291.1\|size8446 | 858 | 105.345 | 4 |
| scaffold79145.1\|size6893 | 42 | 105.345 | 4 |
| scaffold266233.1\|size2994 | 349 | 123.057 | 4 |
| scaffold61788.1\|size8416 | 5902 | 130.464 | 4 |
| scaffold133396.1\|size5183 | 866 | 136.446 | 4 |
| scaffold267007.1\|size2453 | 1879 | 140.376 | 4 |
| scaffold14176.1\|size16115 | 5947 | 145.558 | 4 |
| scaffold5818.1\|size22211 | 11396 | 156.07 | 4 |
| scaffold1680059.1\|size130 | 76 | 156.07 | 4 |
| scaffold772015.1\|size417 | 327 | 156.07 | 4 |
| scaffold7051.1\|size19931 | 3164 | 156.07 | 4 |
| scaffold89544.1\|size6360 | 3656 | 156.07 | 4 |
| scaffold230619.1\|size2871 | 2619 | 156.07 | 4 |
| scaffold69753.1\|size7568 | 3668 | 160.714 | 4 |
| scaffold39100.1\|size10230 | 5324 | 164.274 | 4 |
| scaffold14705.1\|size15597 | 10330 | 170.549 | 4 |
| scaffold131209.1\|size4821 | 1897 | 170.549 | 4 |
| scaffold131209.1\|size4821 | 1911 | 170.549 | 4 |
| scaffold77386.1\|size6988 | 4236 | 179.085 | 4 |
| scaffold77386.1\|size6988 | 4240 | 179.085 | 4 |
| scaffold38404.1\|size11157 | 2051 | 188.432 | 4 |
| scaffold26219.1\|size12331 | 3182 | 197.685 | 4 |
| scaffold26219.1\|size12331 | 3218 | 197.685 | 4 |
| scaffold3294.1\|size24758 | 17673 | 206.32 | 4 |
| scaffold824848.1\|size277 | 176 | 206.32 | 4 |
| scaffold2781.1\|size25790 | 19684 | 214.457 | 4 |
| scaffold17525.1\|size14599 | 7720 | 214.457 | 4 |
| scaffold96908.1\|size6029 | 1078 | 227.715 | 4 |
| scaffold96908.1\|size6029 | 1079 | 227.715 | 4 |
| scaffold96908.1\|size6029 | 1105 | 227.715 | 4 |
| scaffold304897.1\|size2090 | 1168 | 0 | 5 |
| scaffold209905.1\|size3154 | 452 | 17.884 | 5 |
| scaffold209905.1\|size3154 | 407 | 17.884 | 5 |
| scaffold544530.1\|size738 | 81 | 30.4 | 5 |
| scaffold232761.1\|size2835 | 1264 | 43.584 | 5 |
| scaffold1590434.1\|size134 | 54 | 49.46 | 5 |
| scaffold827203.1\|size274 | 44 | 56.145 | 5 |
| scaffold827203.1\|size274 | 66 | 56.145 | 5 |
| scaffold827203.1\|size274 | 96 | 56.145 | 5 |
| scaffold43731.1\|size9666 | 6610 | 62.642 | 5 |
| scaffold13665.1\|size16231 | 12518 | 67.196 | 5 |
| scaffold14666.1\|size15611 | 11037 | 67.196 | 5 |
| scaffold15961.1\|size15115 | 6945 | 67.196 | 5 |
| scaffold15961.1\|size15115 | 6963 | 67.196 | 5 |
| scaffold15961.1\|size15115 | 7002 | 67.196 | 5 |
| scaffold20968.1\|size13570 | 9788 | 67.196 | 5 |
| scaffold33108.1\|size11098 | 6657 | 67.196 | 5 |
| scaffold46186.1\|size9402 | 5477 | 67.196 | 5 |
| scaffold46186.1\|size9402 | 5511 | 67.196 | 5 |
| scaffold217457.1\|size3058 | 464 | 67.196 | 5 |
| scaffold260036.1\|size2529 | 2782 | 67.196 | 5 |
| scaffold274912.1\|size2878 | 893 | 67.196 | 5 |
| scaffold289477.1\|size2187 | 53 | 67.196 | 5 |
| scaffold289477.1\|size2187 | 82 | 67.196 | 5 |
| scaffold289477.1\|size2187 | 100 | 67.196 | 5 |
| scaffold515641.1\|size959 | 179 | 67.196 | 5 |
| scaffold92852.1\|size6202 | 820 | 70.996 | 5 |
| scaffold17529.1\|size14598 | 7274 | 73.937 | 5 |
| scaffold16255.1\|size15022 | 2742 | 79.394 | 5 |
| scaffold232761.1\|size2835 | 1212 | 79.394 | 5 |
| scaffold165558.1\|size3969 | 2143 | 83.593 | 5 |
| scaffold616360.1\|size659 | 59 | 91.305 | 5 |
| scaffold616360.1\|size659 | 98 | 91.305 | 5 |
| scaffold971650.1\|size197 | 80 | 97.374 | 5 |
| scaffold54427.1\|size8781 | 693 | 103.848 | 5 |
| scaffold84656.1\|size6602 | 2943 | 103.848 | 5 |
| scaffold84656.1\|size6602 | 2959 | 103.848 | 5 |
| scaffold84656.1\|size6602 | 2965 | 103.848 | 5 |
| scaffold769587.1\|size1304 | 95 | 103.848 | 5 |
| scaffold1991444.1\|size114 | 102 | 110.747 | 5 |
| scaffold305986.1\|size2084 | 770 | 113.545 | 5 |
| scaffold305986.1\|size2084 | 773 | 113.545 | 5 |
| scaffold304897.1\|size2090 | 1234 | 119.626 | 5 |
| scaffold19759.1\|size13902 | 2042 | 125.657 | 5 |
| scaffold24198.1\|size12768 | 1870 | 125.657 | 5 |
| scaffold95081.1\|size6107 | 1921 | 125.657 | 5 |
| scaffold95081.1\|size6107 | 1945 | 125.657 | 5 |
| scaffold199311.1\|size3329 | 848 | 128.916 | 5 |
| scaffold5906.1\|size40267 | 14466 | 135.668 | 5 |
| scaffold5906.1\|size40267 | 14477 | 135.668 | 5 |
| scaffold5906.1\|size40267 | 14511 | 135.668 | 5 |
| scaffold26862.1\|size12208 | 4484 | 135.668 | 5 |
| scaffold26862.1\|size12208 | 4519 | 135.668 | 5 |
| scaffold26862.1\|size12208 | 4520 | 135.668 | 5 |
| scaffold91066.1\|size6286 | 1857 | 135.668 | 5 |
| scaffold72425.1\|size7283 | 1203 | 142.204 | 5 |
| scaffold14044.1\|size15837 | 4128 | 150.82 | 5 |
| scaffold20846.1\|size13599 | 9703 | 150.82 | 5 |
| scaffold11250.1\|size17107 | 396 | 158.363 | 5 |
| scaffold18860.1\|size14168 | 8196 | 158.363 | 5 |
| scaffold32781.1\|size11407 | 8937 | 168.975 | 5 |
| scaffold21430.1\|size13450 | 6639 | 181.689 | 5 |
| scaffold21430.1\|size13450 | 6652 | 181.689 | 5 |
| scaffold199230.1\|size3330 | 771 | 191.259 | 5 |
| scaffold199230.1\|size3330 | 772 | 191.259 | 5 |
| scaffold6326.1\|size20589 | 15119 | 201.878 | 5 |
| scaffold245870.1\|size2662 | 917 | 201.878 | 5 |
| scaffold537045.1\|size3788 | 903 | 213.459 | 5 |
| scaffold537045.1\|size3788 | 904 | 213.459 | 5 |
| scaffold1167373.1\|size163 | 69 | 0 | 6 |
| scaffold1167373.1\|size163 | 126 | 0 | 6 |
| scaffold39210.1\|size10215 | 8207 | 13.905 | 6 |
| scaffold39210.1\|size10215 | 8259 | 13.905 | 6 |
| scaffold35590.1\|size10723 | 5321 | 19.617 | 6 |
| scaffold9850.1\|size17896 | 6904 | 24.218 | 6 |
| scaffold466291.1\|size1136 | 219 | 24.218 | 6 |
| scaffold74440.1\|size7161 | 478 | 39.583 | 6 |
| scaffold74440.1\|size7161 | 479 | 39.583 | 6 |
| scaffold523.1\|size37022 | 18408 | 56.897 | 6 |
| scaffold4934.1\|size22116 | 14972 | 65.997 | 6 |
| scaffold4934.1\|size22116 | 14980 | 65.997 | 6 |
| scaffold4934.1\|size22116 | 15011 | 65.997 | 6 |
| scaffold60880.1\|size8266 | 678 | 75.29 | 6 |
| scaffold60880.1\|size8266 | 690 | 75.29 | 6 |
| scaffold75564.1\|size7095 | 2756 | 83.827 | 6 |
| scaffold148355.1\|size7408 | 5011 | 95.306 | 6 |
| scaffold2080807.1\|size110 | 84 | 103.305 | 6 |
| scaffold144433.1\|size4733 | 818 | 110.754 | 6 |
| scaffold15651.1\|size15235 | 10818 | 114.258 | 6 |
| scaffold275032.1\|size2337 | 1813 | 120.971 | 6 |
| scaffold119843.1\|size5164 | 3662 | 135.212 | 6 |
| scaffold119843.1\|size5164 | 3680 | 135.212 | 6 |
| scaffold119843.1\|size5164 | 3707 | 135.212 | 6 |
| scaffold234429.1\|size2809 | 962 | 147.376 | 6 |
| scaffold10538.1\|size18091 | 1223 | 147.376 | 6 |
| scaffold79589.1\|size6868 | 4724 | 154.149 | 6 |
| scaffold10359.1\|size17587 | 11930 | 167.764 | 6 |
| scaffold237445.1\|size5504 | 5594 | 167.764 | 6 |
| scaffold50418.1\|size8969 | 7106 | 191.393 | 6 |
| scaffold50418.1\|size8969 | 7123 | 191.393 | 6 |
| scaffold50418.1\|size8969 | 7176 | 191.393 | 6 |
| scaffold13129.1\|size16229 | 13585 | 0 | 7 |
| scaffold37558.1\|size10440 | 5690 | 6.297 | 7 |
| scaffold28998.1\|size11798 | 2188 | 15.045 | 7 |
| scaffold2222091.1\|size105 | 62 | 26.861 | 7 |
| scaffold5371.1\|size21572 | 9953 | 32.197 | 7 |
| scaffold118417.1\|size5211 | 1156 | 36.64 | 7 |
| scaffold23408.1\|size16298 | 6938 | 36.64 | 7 |
| scaffold28538.1\|size11881 | 1521 | 36.64 | 7 |
| scaffold28538.1\|size11881 | 1578 | 36.64 | 7 |
| scaffold113182.1\|size5394 | 2373 | 41.296 | 7 |
| scaffold89099.1\|size7467 | 721 | 47.683 | 7 |
| scaffold1564638.1\|size135 | 74 | 51.829 | 7 |
| scaffold20393.1\|size13725 | 2072 | 51.829 | 7 |
| scaffold866471.1\|size510 | 51 | 59.086 | 7 |
| scaffold2134897.1\|size108 | 62 | 65.111 | 7 |
| scaffold6011.1\|size20913 | 9980 | 70.065 | 7 |
| scaffold17515.1\|size14602 | 1792 | 75.345 | 7 |
| scaffold115062.1\|size8048 | 610 | 80.724 | 7 |
| scaffold2686.1\|size27013 | 21501 | 86.406 | 7 |
| scaffold9016.1\|size22884 | 15712 | 89.358 | 7 |
| scaffold102754.1\|size6330 | 5893 | 89.358 | 7 |
| scaffold102754.1\|size6330 | 5896 | 89.358 | 7 |
| scaffold499846.1\|size1373 | 92 | 93.114 | 7 |
| scaffold7270.1\|size19752 | 511 | 96.872 | 7 |
| scaffold7443.1\|size19605 | 3915 | 96.872 | 7 |
| scaffold47053.1\|size9308 | 1733 | 96.872 | 7 |
| scaffold218556.1\|size3044 | 2204 | 96.872 | 7 |
| scaffold2686.1\|size27013 | 21509 | 102.854 | 7 |
| scaffold75074.1\|size7123 | 1915 | 102.854 | 7 |
| scaffold158810.1\|size4111 | 81 | 114.876 | 7 |
| scaffold137240.1\|size4644 | 2243 | 114.876 | 7 |
| scaffold137240.1\|size4644 | 2294 | 114.876 | 7 |
| scaffold78303.1\|size10252 | 6344 | 125.079 | 7 |
| scaffold161608.1\|size4442 | 2036 | 132.966 | 7 |
| scaffold116890.1\|size5266 | 2748 | 146.116 | 7 |
| scaffold176459.1\|size3734 | 819 | 153.194 | 7 |
| scaffold84613.1\|size8226 | 2770 | 0 | 8 |
| scaffold7202.1\|size19812 | 6834 | 7.031 | 8 |
| scaffold7202.1\|size19812 | 6870 | 7.031 | 8 |
| scaffold7202.1\|size19812 | 6880 | 7.031 | 8 |
| scaffold13586.1\|size19593 | 5297 | 11.043 | 8 |
| scaffold64060.1\|size8219 | 4602 | 15.649 | 8 |
| scaffold68162.1\|size7558 | 1783 | 20.937 | 8 |
| scaffold219119.1\|size3038 | 236 | 20.937 | 8 |
| scaffold131557.1\|size4811 | 1811 | 33.239 | 8 |
| scaffold5956.1\|size20965 | 1092 | 42.576 | 8 |
| scaffold894.1\|size33222 | 22728 | 50.503 | 8 |
| scaffold68972.1\|size7500 | 2425 | 57.915 | 8 |
| scaffold160045.1\|size4084 | 454 | 63.306 | 8 |
| scaffold160045.1\|size4084 | 470 | 63.306 | 8 |
| scaffold117810.1\|size5232 | 1414 | 70.186 | 8 |
| scaffold367316.1\|size1626 | 348 | 78.01 | 8 |
| scaffold367316.1\|size1626 | 354 | 78.01 | 8 |
| scaffold367316.1\|size1626 | 395 | 78.01 | 8 |
| scaffold367316.1\|size1626 | 417 | 78.01 | 8 |
| scaffold15241.1\|size15389 | 3186 | 86.631 | 8 |
| scaffold226991.1\|size3847 | 581 | 94.562 | 8 |
| scaffold244926.1\|size3202 | 2531 | 94.562 | 8 |
| scaffold10615.1\|size17447 | 10132 | 101.437 | 8 |
| scaffold562048.1\|size707 | 85 | 111.833 | 8 |
| scaffold90949.1\|size6292 | 4163 | 119.217 | 8 |
| scaffold68972.1\|size7500 | 2471 | 126.557 | 8 |
| scaffold160045.1\|size4084 | 475 | 132.253 | 8 |
| scaffold160045.1\|size4084 | 513 | 132.253 | 8 |
| scaffold560675.1\|size709 | 385 | 0 | 9 |
| scaffold961176.1\|size199 | 129 | 4.889 | 9 |
| scaffold1977.1\|size28740 | 8180 | 12.29 | 9 |
| scaffold157309.1\|size5323 | 3129 | 17.103 | 9 |
| scaffold112116.1\|size5432 | 2383 | 22.356 | 9 |
| scaffold104248.1\|size7012 | 2597 | 26.603 | 9 |
| scaffold20836.1\|size13602 | 8338 | 34.793 | 9 |
| scaffold11235.1\|size17112 | 10449 | 41.538 | 9 |
| scaffold401305.1\|size1415 | 1250 | 62.008 | 9 |
| scaffold401305.1\|size1415 | 1298 | 62.008 | 9 |
| scaffold831380.1\|size936 | 76 | 71.445 | 9 |
| scaffold255335.1\|size2958 | 2618 | 75.924 | 9 |
| scaffold24822.1\|size12637 | 7075 | 80.087 | 9 |
| scaffold5204.1\|size21789 | 16366 | 88.036 | 9 |
| scaffold125329.1\|size4996 | 599 | 93.107 | 9 |
| scaffold155180.1\|size4190 | 590 | 101.79 | 9 |
| scaffold179063.1\|size8191 | 1362 | 112.328 | 9 |
| scaffold255092.1\|size2575 | 352 | 0 | 10 |
| scaffold119195.1\|size5185 | 701 | 4.532 | 10 |
| scaffold46688.1\|size11229 | 8438 | 12.528 | 10 |
| scaffold764483.1\|size1311 | 211 | 17.437 | 10 |
| scaffold788938.1\|size1045 | 864 | 21.797 | 10 |
| scaffold71495.1\|size8256 | 5647 | 29.083 | 10 |
| scaffold20257.1\|size14273 | 3836 | 43.642 | 10 |
| scaffold20257.1\|size14273 | 3855 | 43.642 | 10 |
| scaffold9051.1\|size18417 | 5052 | 52.931 | 10 |
| scaffold94411.1\|size6133 | 1090 | 59.312 | 10 |
| scaffold218420.1\|size3046 | 2015 | 59.312 | 10 |
| scaffold620706.1\|size656 | 353 | 59.312 | 10 |
| scaffold3354.1\|size24596 | 4941 | 65.248 | 10 |
| scaffold472228.1\|size1256 | 607 | 68.256 | 10 |
| scaffold55479.1\|size8514 | 4495 | 73.535 | 10 |
| scaffold55479.1\|size8514 | 4496 | 73.535 | 10 |
| scaffold55479.1\|size8514 | 4517 | 73.535 | 10 |
| scaffold55479.1\|size8514 | 4518 | 73.535 | 10 |
| scaffold55479.1\|size8514 | 4521 | 73.535 | 10 |
| scaffold21556.1\|size20098 | 13070 | 79.052 | 10 |
| scaffold68207.1\|size7555 | 2365 | 86.596 | 10 |
| scaffold11955.1\|size16746 | 10702 | 86.596 | 10 |
| scaffold391708.1\|size1523 | 155 | 88.007 | 10 |
| scaffold26888.1\|size12448 | 5022 | 102.997 | 10 |
| scaffold154012.1\|size4218 | 635 | 102.997 | 10 |
| scaffold419982.1\|size4274 | 600 | 0 | 11 |
| scaffold419982.1\|size4274 | 612 | 0 | 11 |
| scaffold345459.1\|size1717 | 347 | 16.087 | 11 |
| scaffold42174.1\|size9849 | 5567 | 26.638 | 11 |
| scaffold42174.1\|size9849 | 5591 | 26.638 | 11 |
| scaffold180904.1\|size3648 | 1007 | 35.974 | 11 |
| scaffold1383169.1\|size145 | 85 | 42.832 | 11 |
| scaffold23825.1\|size12853 | 8838 | 49.847 | 11 |
| scaffold133591.1\|size5348 | 2125 | 62.461 | 11 |
| scaffold133591.1\|size5348 | 2126 | 62.461 | 11 |
| scaffold133591.1\|size5348 | 2114 | 62.461 | 11 |
| scaffold148684.1\|size4352 | 2841 | 68.574 | 11 |
| scaffold148684.1\|size4352 | 2844 | 68.574 | 11 |
| scaffold148684.1\|size4352 | 2862 | 68.574 | 11 |
| scaffold148684.1\|size4352 | 2892 | 68.574 | 11 |
| scaffold38788.1\|size10276 | 6534 | 71.389 | 11 |
| scaffold62496.1\|size8095 | 5306 | 74.122 | 11 |
| scaffold62496.1\|size8095 | 5349 | 79.481 | 11 |
| scaffold628450.1\|size651 | 44 | 0 | 12 |
| scaffold628450.1\|size651 | 84 | 0 | 12 |
| scaffold17608.1\|size14573 | 323 | 17.901 | 12 |
| scaffold140100.1\|size8324 | 1480 | 23.188 | 12 |
| scaffold26580.1\|size12263 | 4731 | 27.619 | 12 |
| scaffold55168.1\|size9271 | 3986 | 27.619 | 12 |
| scaffold1504449.1\|size138 | 61 | 40.101 | 12 |
| scaffold1244148.1\|size155 | 44 | 47.068 | 12 |
| scaffold1522041.1\|size137 | 99 | 50.162 | 12 |
| scaffold194391.1\|size4289 | 3494 | 53.085 | 12 |
| scaffold1678496.1\|size130 | 98 | 59.465 | 12 |
| scaffold408097.1\|size1325 | 79 | 63.094 | 12 |
| scaffold138521.1\|size5660 | 1502 | 0 | 13 |
| scaffold95450.1\|size6293 | 4732 | 4.224 | 13 |
| scaffold95450.1\|size6293 | 4733 | 4.224 | 13 |
| scaffold81948.1\|size6742 | 4652 | 12.267 | 13 |
| scaffold195443.1\|size3990 | 2045 | 22.259 | 13 |
| scaffold195443.1\|size3990 | 2046 | 26.597 | 13 |
| scaffold103301.1\|size5764 | 933 | 39.698 | 13 |
| scaffold103301.1\|size5764 | 934 | 39.698 | 13 |
| scaffold23303.1\|size12976 | 452 | 0 | 14 |
| scaffold580841.1\|size686 | 452 | 3.454 | 14 |
| scaffold68818.1\|size7512 | 4849 | 3.454 | 14 |
| scaffold68818.1\|size7512 | 4920 | 3.454 | 14 |
| scaffold25920.1\|size12396 | 8033 | 8.745 | 14 |
| scaffold25920.1\|size12396 | 8036 | 8.745 | 14 |
| scaffold1066.1\|size32102 | 21857 | 16.486 | 14 |
| scaffold109609.1\|size5522 | 4325 | 20.775 | 14 |
| scaffold107853.1\|size5824 | 46 | 31.226 | 14 |
| scaffold232738.1\|size2835 | 809 | 35.561 | 14 |
| scaffold327319.1\|size1885 | 949 | 38.019 | 14 |
| scaffold327319.1\|size1885 | 951 | 38.019 | 14 |
| scaffold327319.1\|size1885 | 952 | 38.019 | 14 |
| scaffold327319.1\|size1885 | 953 | 38.019 | 14 |
| scaffold278539.1\|size2290 | 1124 | 38.019 | 14 |
| scaffold365769.1\|size5405 | 1659 | 0 | 15 |
| scaffold661387.1\|size633 | 491 | 1.689 | 15 |
| scaffold661387.1\|size633 | 506 | 1.689 | 15 |
| scaffold235358.1\|size2794 | 2040 | 13.128 | 15 |
| scaffold21465.1\|size13438 | 3229 | 17.421 | 15 |
| scaffold21465.1\|size13438 | 3211 | 25.099 | 15 |
| scaffold235358.1\|size2794 | 2036 | 30.083 | 15 |
| scaffold826013.1\|size276 | 130 | 0 | 16 |
| scaffold826013.1\|size276 | 184 | 0 | 16 |
| scaffold212269.1\|size3860 | 2308 | 5.504 | 16 |
| scaffold212269.1\|size3860 | 2317 | 5.504 | 16 |
| scaffold261335.1\|size2951 | 1484 | 10.701 | 16 |
| scaffold1946.1\|size28097 | 9947 | 13.983 | 16 |
| scaffold1190256.1\|size161 | 51 | 18.067 | 16 |
| scaffold36442.1\|size11962 | 6798 | 23.49 | 16 |
| scaffold262433.1\|size2506 | 133 | 23.49 | 16 |
| scaffold100007.1\|size5898 | 2004 | 0 | 17 |
| scaffold40261.1\|size10081 | 1047 | 5.647 | 17 |
| scaffold770534.1\|size3331 | 2575 | 10.198 | 17 |
| scaffold7754.1\|size19324 | 5728 | 16.359 | 17 |
| scaffold3730.1\|size23950 | 14955 | 19.906 | 17 |
| scaffold62996.1\|size15491 | 1960 | 0 | 18 |
| scaffold62996.1\|size15491 | 2004 | 0 | 18 |
| scaffold80085.1\|size6840 | 5555 | 8.129 | 18 |
| scaffold1617.1\|size29377 | 8676 | 16.587 | 18 |
| scaffold117940.1\|size5228 | 4306 | 19.3 | 18 |
| scaffold117940.1\|size5228 | 4362 | 19.3 | 18 |
| scaffold248777.1\|size2633 | 354 | 0 | 19 |
| scaffold17000.1\|size14772 | 330 | 3.965 | 19 |
| scaffold33479.1\|size13603 | 672 | 3.965 | 19 |
| scaffold147383.1\|size4386 | 3382 | 3.965 | 19 |
| scaffold148227.1\|size4365 | 2625 | 3.965 | 19 |
| scaffold169954.1\|size3877 | 2108 | 3.965 | 19 |
| scaffold169954.1\|size3877 | 2110 | 3.965 | 19 |
| scaffold318692.1\|size1993 | 212 | 14.031 | 19 |
| scaffold318692.1\|size1993 | 252 | 14.031 | 19 |

**Table S4**: Social status and boldness phenotype for each individual in the dataset. Individual codes include locality (BL=Boone Lake, CH=Chilhowee Dam, MH=Melton Hill, ML=Melton Lake), the nests number, and the individual number. Individuals that died prematurely or that had low quality sequencing results are excluded from this list. Solitary individuals receive a social score of 0, while individuals collected from social groups receive a score of 1. Boldness is scored based on the number of seconds that a spider remains in a death feign position (longer duration is associated with shy individuals, while a faster recovery is observed in bold individuals).

| individual_id | social | boldness |
| --- | --- | --- |
| BL_1_1 | 0 | 0 |
| BL_12_2 | 1 | 39 |
| BL_12_4 | 1 | 25 |
| BL_12_5 | 1 | 21 |
| BL_14_11 | 1 | 82 |
| BL_14_21 | 1 | 56 |
| BL_14_23 | 1 | 0 |
| BL_14_2 | 1 | 23 |
| BL_14_3 | 1 | 34 |
| BL_14_5 | 1 | 41 |
| BL_15_1 | 0 | 0 |
| BL_19_1 | 1 | 2 |
| BL_19_4 | 1 | NA |
| BL_20_1 | 0 | 59 |
| BL_21_1 | 0 | 11 |
| BL_2_1 | 0 | 37 |
| BL_22_1 | 0 | 24 |
| BL_23_1 | 0 | 107 |
| BL_24_1 | 0 | 2 |
| BL_25_1 | 0 | 33 |
| BL_26_1 | 0 | 4 |
| BL_27_1 | 1 | 41 |
| BL_27_2 | 1 | 0 |
| BL_27_3 | 1 | 54 |
| BL_27_4 | 1 | 0 |
| BL_27_5 | 1 | 39 |
| BL_29_1 | 1 | 124 |
| BL_29_2 | 1 | 8 |
| BL_30_1 | 0 | 35 |
| BL_31_1 | 0 | 34 |
| BL_3_1 | 1 | 0 |
| BL_32_3 | 1 | 46 |
| BL_32_4 | 1 | 20 |
| BL_32_5 | 1 | 5 |
| BL_3_2 | 1 | 0 |
| BL_33_1 | 0 | 4 |
| BL_3_3 | 1 | 0 |
| BL_3_4 | 1 | 80 |
| BL_3_5 | 1 | 0 |
| BL_4_1 | 1 | 0 |
| BL_4_2_Male | 1 | 5 |
| BL_4_3 | 1 | 4 |
| BL_4_4_Male | 1 | 53 |
| BL_4_5 | 1 | 0 |
| BL_5_1 | 1 | 6 |
| BL_5_2 | 1 | 0 |
| BL_6_2 | 1 | 38 |
| BL_6_3 | 1 | 41 |
| BL_6_5 | 1 | 43 |
| BL_7_1 | 0 | 77 |
| BL_8_1 | 0 | 79 |
| CH_10_1 | 1 | 16 |
| CH_10_2 | 1 | 65 |
| CH_10_3 | 1 | 20 |
| CH_10_4 | 1 | 74 |
| CH_10_5 | 1 | 24 |
| CH_11_1 | 1 | 28 |
| CH_11_2 | 1 | 3 |
| CH_1_1 | 0 | 0 |
| CH_12_1 | 0 | 43 |
| CH_13_1 | 0 | 6 |
| CH_14_1 | 0 | 215 |
| CH_15_1 | 0 | 0 |
| CH_16_1 | 0 | 21 |
| CH_17_1 | 0 | 27 |
| CH_18_1 | 0 | 2 |
| CH_19_1 | 0 | 0 |
| CH_20_1 | 0 | 0 |
| CH_21_1 | 1 | 59 |
| CH_21_2 | 1 | 204 |
| CH_21_3 | 1 | 15 |
| CH_21_4 | 1 | 30 |
| CH_21_5 | 1 | 0 |
| CH_22_1 | 0 | 1 |
| CH_23_1 | 1 | 38 |
| CH_23_4 | 1 | 7 |
| CH_23_5 | 1 | 20 |
| CH_23A_2 | 1 | NA |
| CH_23B_2 | 1 | NA |
| CH_24_1 | 0 | 13 |
| CH_25_1 | 0 | 69 |
| CH_26_1 | 0 | 106 |
| CH_27_1 | 0 | 0 |
| CH_28_1 | 0 | 87 |
| CH_29_1 | 1 | 70 |
| CH_29_2 | 1 | 16 |
| CH_29_3 | 1 | 122 |
| CH_29_4 | 1 | 60 |
| CH_30_1 | 1 | 45 |
| CH_30_2 | 1 | 35 |
| CH_30_3 | 1 | 0 |
| CH_30_4 | 1 | 16 |
| CH_31_1 | 1 | 0 |
| CH_31_2 | 1 | 41 |
| CH_3_1 | 0 | 368 |
| CH_33_1 | 1 | 41 |
| CH_4_1 | 0 | 71 |
| CH_5_1 | 0 | 27 |
| CH_6_1 | 0 | 132 |
| CH_7_1 | 1 | 35 |
| CH_7_2 | 1 | 46 |
| CH_7_3 | 1 | 12 |
| CH_7_4 | 1 | 139 |
| CH_7_5 | 1 | 17 |
| CH_8_1 | 1 | 63 |
| CH_8_2 | 1 | 31 |
| MH_11_1 | 0 | 10 |
| MH_1_1 | 0 | 20 |
| MH_12_4 | 1 | 6 |
| MH_13_10 | 1 | 59 |
| MH_13_11 | 1 | 0 |
| MH_13_1 | 1 | 0 |
| MH_13_2 | 1 | 19 |
| MH_13_3 | 1 | 0 |
| MH_13_4 | 1 | 15 |
| MH_13_9 | 1 | 0 |
| MH_15_1 | 0 | 0 |
| MH_16_10 | 1 | 0 |
| MH_16_1 | 1 | 73 |
| MH_16_4 | 1 | 0 |
| MH_16_5 | 1 | 26 |
| MH_16_7 | 1 | 7 |
| MH_16_9 | 1 | 0 |
| MH_17_4 | 1 | 25 |
| MH_17_5 | 1 | 0 |
| MH_18_1 | 0 | 231 |
| MH_19_1 | 1 | 29 |
| MH_19_3 | 1 | 0 |
| MH_19_5 | 1 | 0 |
| MH_20_57 | 1 | NA |
| MH_20_66 | 1 | 4 |
| MH_20_69 | 1 | 62 |
| MH_20_71 | 1 | 5 |
| MH_20_75 | 1 | 75 |
| MH_21_1 | 1 | 148 |
| MH_21_2 | 1 | 27 |
| MH_21_3 | 1 | 18 |
| MH_21_4 | 1 | 34 |
| MH_21_5 | 1 | 11 |
| MH_23_1 | 0 | 2 |
| MH_24_2 | 1 | 7 |
| MH_24_3 | 1 | 1 |
| MH_24_4 | 1 | 5 |
| MH_25_1 | 1 | 10 |
| MH_25_2 | 1 | 52 |
| MH_26_1 | 0 | 78 |
| MH_27_4 | 1 | 119 |
| MH_29_1 | 1 | 0 |
| MH_29_3 | 1 | 8 |
| MH_4_1 | 0 | 4 |
| MH_9_1 | 0 | 77 |
| ML_10_1 | 0 | 0 |
| ML_11_1 | 0 | 79 |
| ML_1_1 | 1 | 116 |
| ML_12_1 | 1 | 0 |
| ML_12_2 | 1 | 0 |
| ML_12_3 | 1 | 0 |
| ML_12_4 | 1 | 0 |
| ML_12_5 | 1 | 0 |
| ML_1_2 | 1 | 0 |
| ML_1_3 | 1 | 29 |
| ML_14_1 | 1 | 15 |
| ML_14_2 | 1 | 0 |
| ML_14_3 | 1 | 0 |
| ML_14_4 | 1 | 19 |
| ML_14_5 | 1 | 22 |
| ML_1_4 | 1 | 0 |
| ML_1_5 | 1 | 8 |
| ML_19_1 | 0 | 11 |
| ML_20_11 | 1 | 0 |
| ML_20_1 | 1 | 54 |
| ML_20_2 | 1 | 0 |
| ML_20_3 | 1 | 8 |
| ML_20_4 | 1 | 0 |
| ML_20_5 | 1 | 0 |
| ML_21_1 | 0 | 5 |
| ML_2_1 | 1 | 118 |
| ML_22_1 | 1 | 0 |
| ML_22_2 | 1 | 4 |
| ML_22_3 | 1 | 3 |
| ML_22_4 | 1 | 63 |
| ML_22_5 | 1 | 0 |
| ML_2_2 | 1 | 0 |
| ML_23_1 | 0 | 38 |
| ML_2_3 | 1 | 24 |
| ML_24_1 | 1 | 47 |
| ML_2_4 | 1 | 116 |
| ML_2_5 | 1 | 31 |
| ML_26_2 | 1 | 35 |
| ML_27_1 | 1 | 37 |
| ML_27_2 | 1 | 134 |
| ML_28_1 | 0 | 22 |
| ML_29_1 | 1 | 15 |
| ML_29_2 | 1 | 11 |
| ML_29_3 | 1 | 1 |
| ML_29_5 | 1 | 1 |
| ML_30_1 | 1 | 70 |
| ML_30_2 | 1 | 5 |
| ML_30_4 | 1 | 1 |
| ML_30_5 | 1 | 324 |
| ML_31_1 | 0 | 2 |
| ML_3_1 | 0 | 76 |
| ML_32_1 | 1 | 86 |
| ML_32_2 | 1 | 6 |
| ML_33_1 | 0 | 67 |
| ML_35_1 | 0 | 25 |
| ML_5_1 | 1 | 1 |
| ML_6_1 | 0 | 0 |
| ML_8_1 | 0 | 1 |
| ML_9_1 | 0 | 12 |
